# Supplementary material for: Biallelic non-productive enhancer-promoter interactions precede imprinted expression of Kcnk9 during mouse neural commitment
Source: HGG Adv. 2024 Jan 30;5(2):100271. doi: 10.1016/j.xhgg.2024.100271 (PMC10869267; doi:10.1016/j.xhgg.2024.100271)
Supplement: Document S1. Figures S1–S6 and Tables S1–S3 [file mmc1.pdf]

HGGA, Volume *5*

Supplemental information

Biallelic non-productive enhancer-promoter interactions precede imprinted expression of *Kcnk9* during mouse neural commitment

Cecilia Rengifo Rojas, Jil Cercy, Sophie Perillous, Céline Gonthier-Guéret, Bertille Montibus, Stéphanie Maupetit-Méhouas, Astrid Espinadel, Marylou Dupré, Charles C. Hong, Kenichiro Hata, Kazuhiko Nakabayashi, Antonius Plagge, Tristan Bouschet, Philippe Arnaud, Isabelle Vaillant, and Franck Court

Supplemental figures and tables :

Figure S1: In the corticogenesis model, expression patterns at the *Peg13* domain recapitulate those observed in embryonic brain *in vivo*

Figure S2: DNA methylation is lost at the *Peg13 DMR* in E9.5 *Dnmt3l* $^{-/+}$ embryos

Figure S3: *Peg13* DMR and *Kcnk9* methylation patterns

Figure S4: Parental allelic 4C-seq signals for the *Peg13* DMR viewpoint in in ESCs and NPCs

Figure S5: The *Peg13* DMR and *PE* interactomes organize the higher-order chromatin conformation at the *Peg13* domain

Figure S6: PE molecular signature dynamics during neural commitment

Table S1: Reagent and Resource

Table S2: Details of the primers used in this study

Table S3: Details of the antibodies used in this study

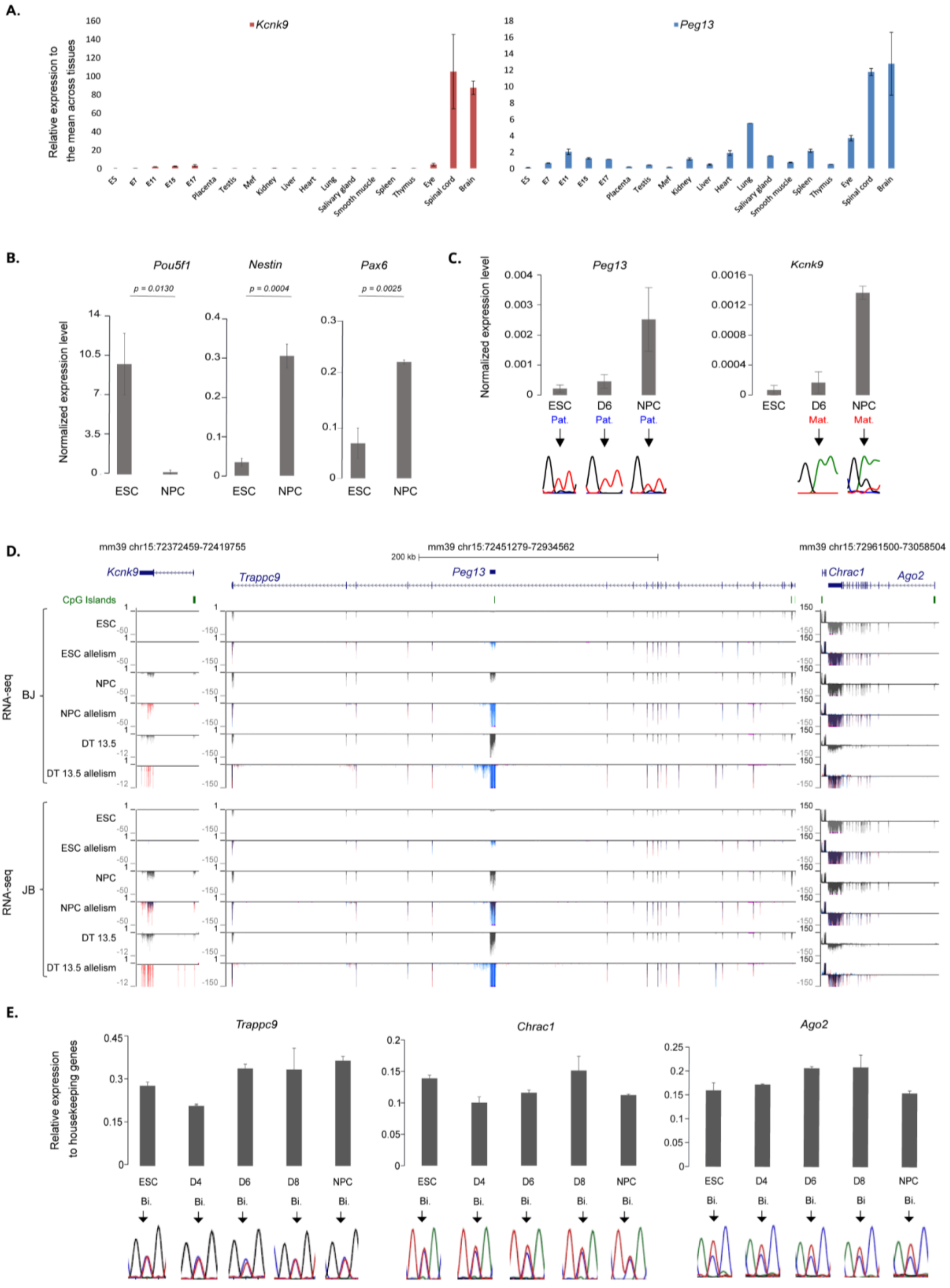

Figure S1: In the corticogenesis model, expression patterns at the *Peg13* domain recapitulate those observed in embryonic brain *in vivo*
A) Microfluidic-based RT-qPCR analysis of *Kcnk9* and *Peg13* expression in the indicated tissues and cell types. Results are presented as the fold enrichment of the mean expression level detected in all tissues, after normalization to the geometric mean of the expression of the three housekeeping genes *Arbp*, *Gapdh* and *Tbp*. Data were from two independent experiments, each analyzed in duplicate. **B)** P*ou5f1*, *Nestin* and *Pax6* expression levels in ESCs (n=6) and in NPCs at day 12 (D12) (n=6) of *in vitro* corticogenesis. P values determined with the unpaired t test. **C)** Quantitative RT-PCR analysis of *Kcnk9* and *Peg13* expression levels in J/B ESCs (n = 3), at D6 (n=3) of *in vitro* corticogenesis and in NPCs (D12; n=3). The parental origin of expression is shown below. **D)** Quantitative RT-PCR analysis of the expression levels of *Trappc9, Chrac1* and *Ago2* in B/J ESCs (*n* = 4) and at the D4 (*n* = 2), D6 (n=2), D8 (*n* = 2), and NPC (D12; *n* = 4) stages of *in vitro* corticogenesis. The parental origin of expression is shown below. **E)** Genome browser view at the *Peg13* domain to show the allelic oriented RNA-seq signal in B/J (upper panel) and J/B (lower panel) ESCs, NPCs and embryonic dorsal telencephalon (DT); re-analyzed data from 21. For each condition the quantitative and the merged allelic RNA-seq signals are shown in the upper and lower panel, respectively. Maternal and paternal expression levels are shown in red and blue, respectively. In **B)** to **D)** results are presented as the percentage of expression relative to the geometric mean of the expression of the three housekeeping genes *Gapdh*, *Gus* and *Tbp*. Data are the mean ± SEM.

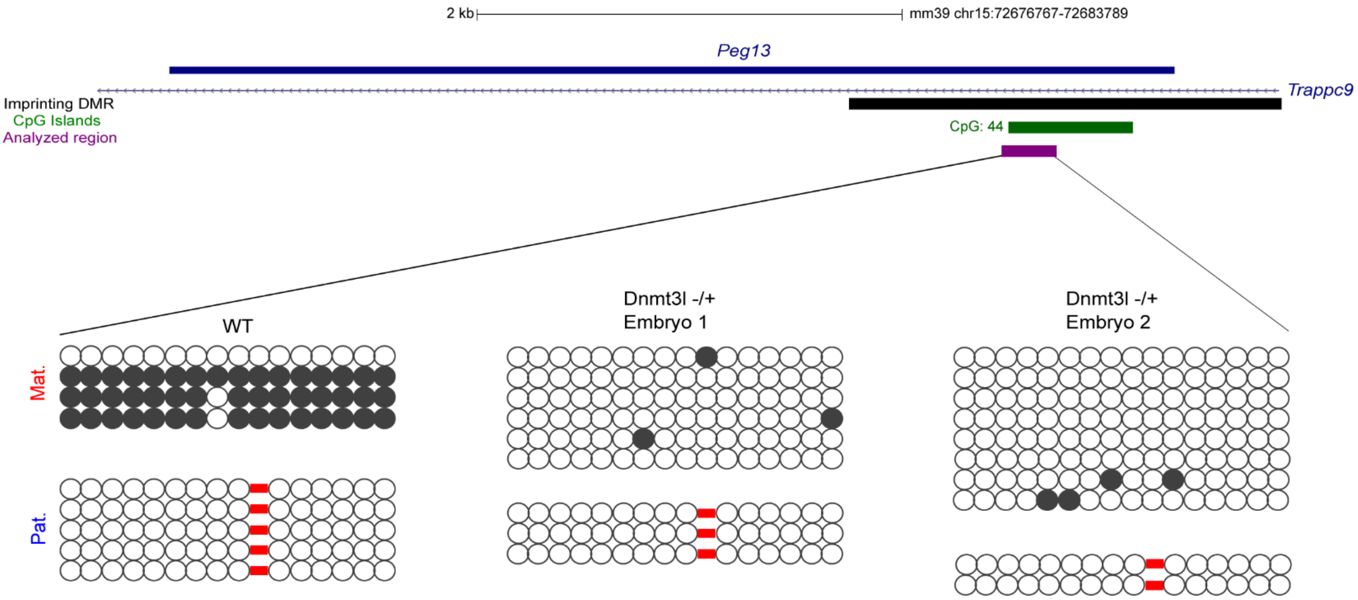

Figure S2: DNA methylation is lost at the *Peg13 DMR* in E9.5 *Dnmt3l* $^{-/+}$ embryos

Map of the mouse *Peg13* locus showing the CpG islands (CGI) as described in the UCSC Genome Browser. The region analyzed by whole-genome bisulfite sequencing is shown in purple. The lower panel shows bisulfite sequencing-derived data from one wild type (WT) and two *Dnmt3l*$^{-/+}$ E9.5 embryos. Each horizontal row of circles represents the CpG dinucleotides on a single chromosome. Solid circles, methylated CpG dinucleotides; open circles, unmethylated CpG dinucleotides. Parental origin (Mat., maternal; Pat., paternal) was determined using strain-specific SNPs. Red rectangles indicate CpGs that are missing due to parental allele specific SNPs.

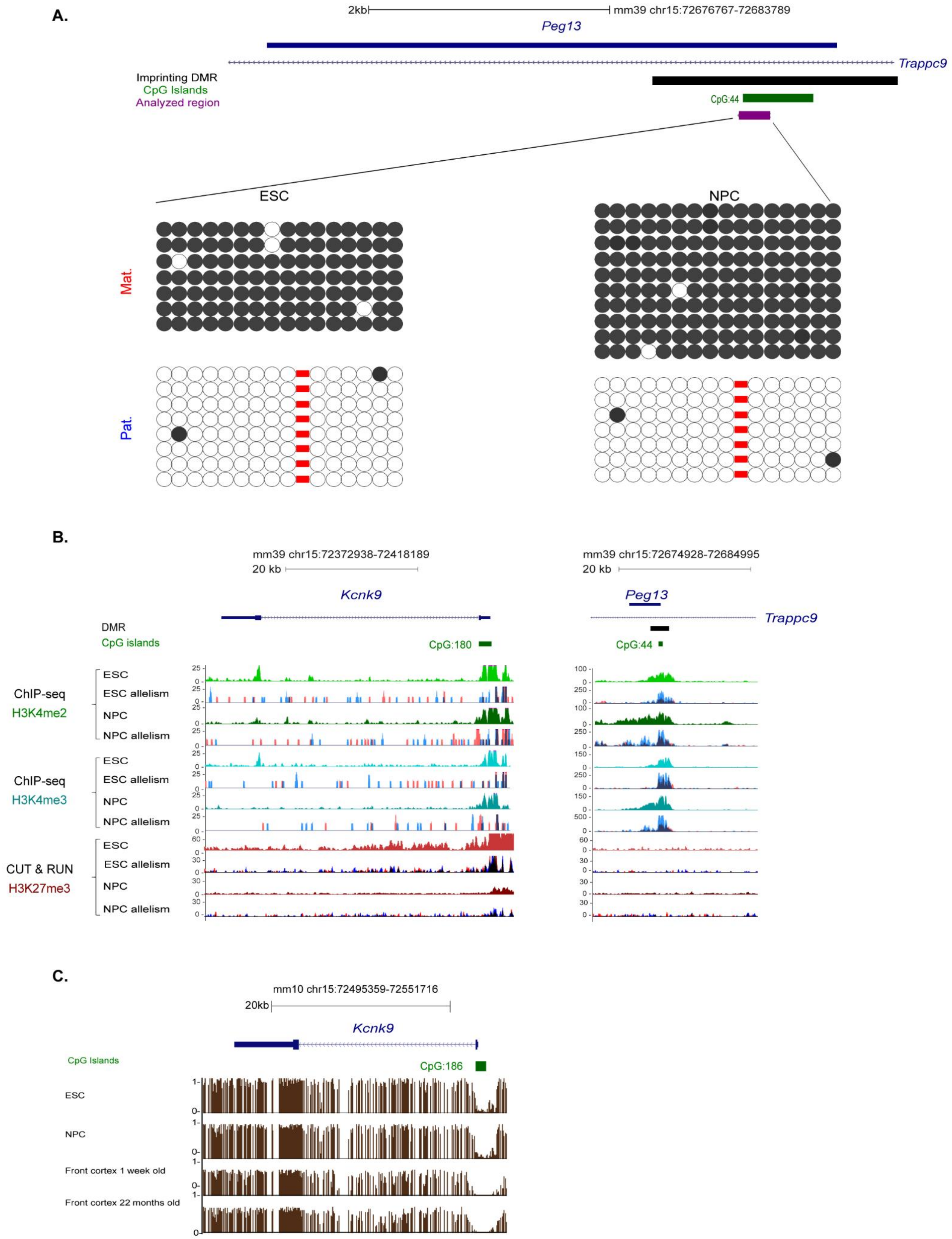

Figure S3: *Peg13* DMR and *Kcnk9* methylation patterns

A) Map of the mouse *Peg13* locus showing the CpG islands (CGI) as described in the UCSC Genome Browser. The region analyzed by bisulfite sequencing is shown in purple. The lower panel shows bisulfite sequencing-derived data from ESCs and NPCs. Each horizontal row of circles represents the CpG dinucleotides on a single chromosome. Solid circles, methylated CpG dinucleotides; open circles, unmethylated CpG dinucleotides. Parental origin (Mat., maternal; Pat., paternal) was determined using strain-specific SNPs. Red rectangles indicate CpGs that are missing due to parental allele specific SNPs. **B)** Genome Browser view at the *Kcnk9* and *Peg13* loci to show CpG island (CGI) positions and H3K4me2, H3K4me3 and H3K27me3 enrichment in J/B ESCs and NPCs. The quantitative and the merged parental allelic signals are shown in the upper and lower panels, respectively. Maternal and paternal enrichments are shown in red and blue, respectively. C) Genome Browser view at the *Kcnk9* locus to show the CpG island (CGI) position and non-allelic WGBS methylation dataset from mouse ESCs, NPCs and frontal cortex.

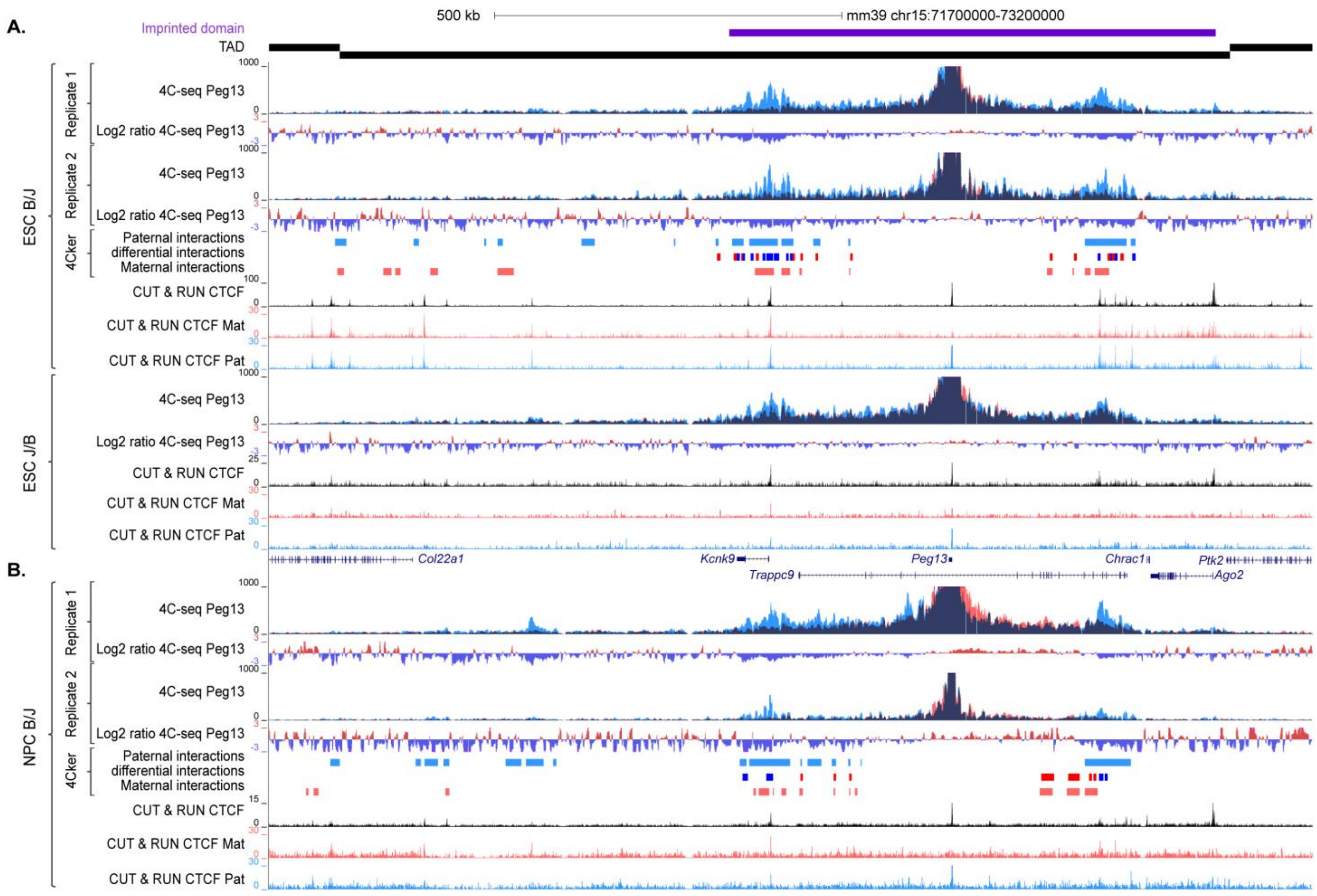

Figure S4: Parental allelic 4C-seq signals for the *Peg13* DMR viewpoint in in ESCs and NPCs

A) Similar parental allelic 4C-seq signals for the *Peg13* DMR viewpoint in B/J and J/B ESCs. Genome browser view at the *Kcnk9* -*Trappc9* region to show B/J (upper panel; n=2) and J/B (lower panel, n=1) ESC allelic 4C-seq data for the *Peg13* DMR viewpoint and CTCF C&R signals. **B**) Parental allelic 4C-seq signals for the *Peg13* DMR viewpoint in duplicate (n=2) in B/J NPCs. In **A)** and **B)** 4C-seq data are shown by merging the allelic signals; contacts mediated by the paternal and maternal alleles are shown in blue and red, respectively. The maternal/paternal interaction ratio is indicated. Paternal (blue), maternal (red) and significant ($p<0.05$) allelic differential interactions, identified with the 4Cker tool, are shown. The relative positions of the TAD and the imprinted domain are shown. *Peg13* DMR interactions are largely confined to the imprinted domain within the TAD.

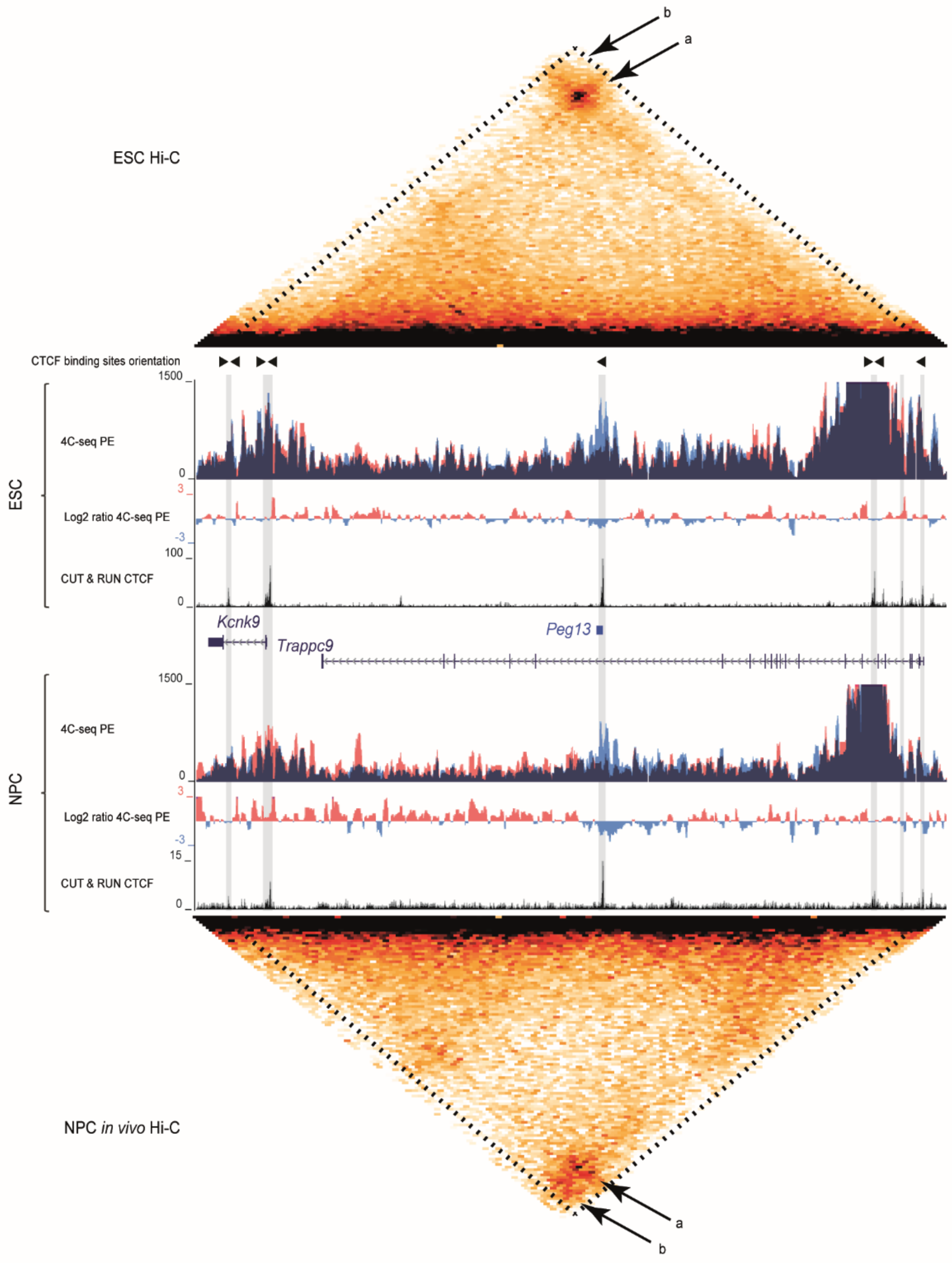

Supp Figure 5: The *Peg13* DMR and *PE* interactomes organize the higher-order chromatin conformation at the *Peg13* domain

Genome Browser view at the *Kcnk9 -Trappc9* region in ESCs (upper panel) and NPCs (bottom panel); re-analyzed Hi-C data, allelic 4C-seq data for the *Peg13* DMR, *Kcnk9* promoter and the PE viewpoints and CTCF C&R signals. 4C-seq data are presented by merging the allelic signals, contacts mediated by the paternal and maternal alleles are shown in blue and red, respectively. The maternal/paternal interaction ratio is indicated where. The sub-TAD containing the *Kcnk9-Peg13* region is delineated by a dotted line; a and b denote the contact between PE and the *Kcnk9* promoter and between PE and the intronic regions, respectively. The orientation of CTCF binding sites is indicated by arrows.

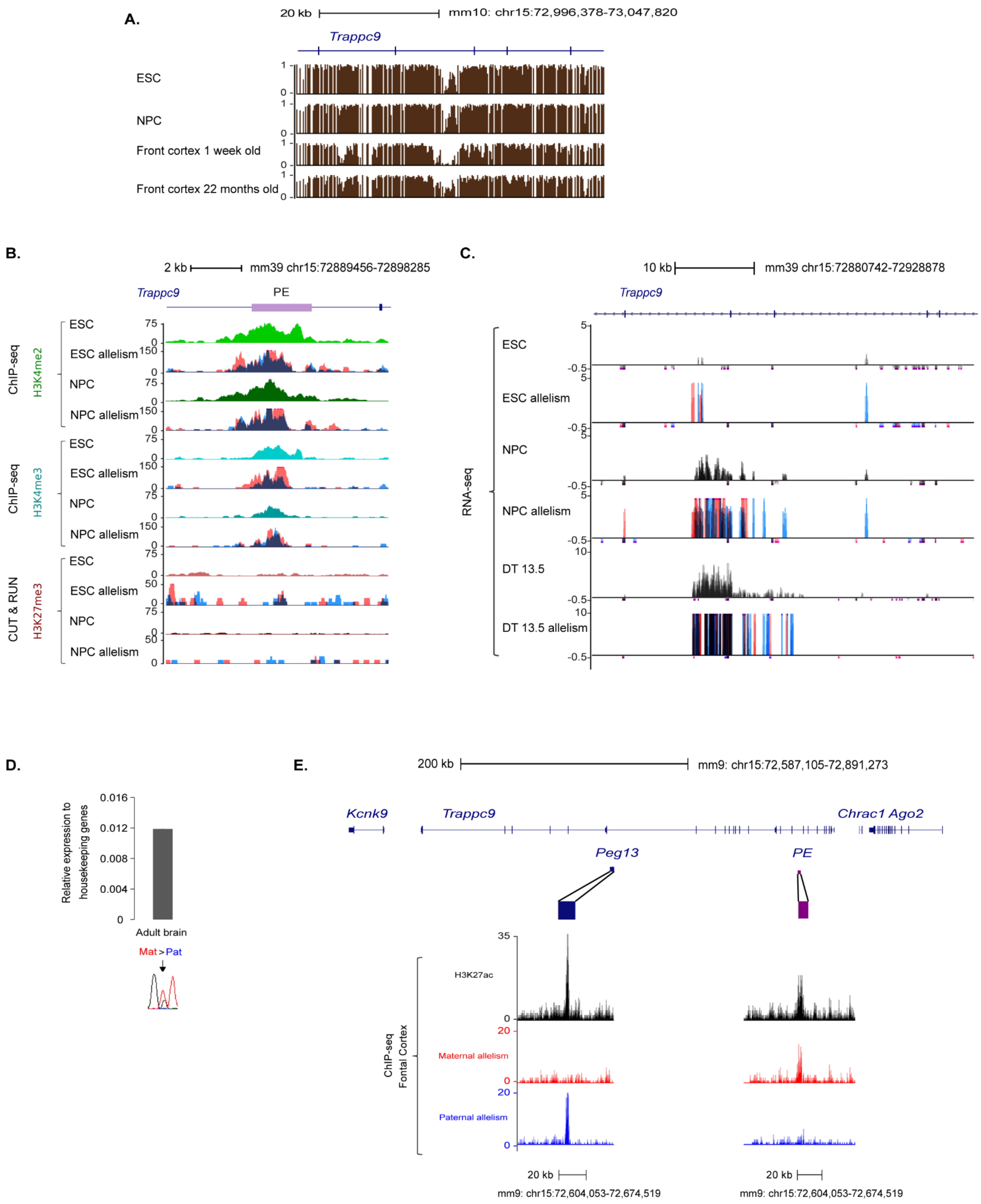

Supp Figure 6: The *Peg13* DMR and *PE* interactomes organize the higher-order chromatin conformation at the *Peg13* domain

A) Genome Browser view at the *PE* region (highlighted in gray) to show non-allelic WGBS methylation data from mouse ESCs, NPCs and frontal cortex. **B)** Genome browser view at the intronic PE region to show ZPF57, H3K4me2, H3K4me3 and H3K27me3 enrichment in J/B ESCs and NPCs. The quantitative and the merged parental allelic signals are shown in the upper and lower panels, respectively. Maternal and paternal enrichments are in red and blue, respectively. **C)** Genome browser view at the PE region to show the allelic oriented RNA-seq signals in J/B ESCs, NPCs and embryonic dorsal telencephalon (DT); re-analyzed data from 21. For each condition, the quantitative and the merged parental allelic RNA-seq signals are shown in the top and bottom panels, respectively. Maternal and paternal expression are in red and blue, respectively. **D)** Quantitative RT-PCR analyses to assess PE-associated eRNA expression in adult brains (n=2). Data are the mean ± SEM. The parental origin of expression is shown below. **E)** Genome Browser view at the *Peg13* and *PE* regions to show allelic H3K27ac enrichment in adult mouse frontal cortex.

**Table S1 :** REAGENT or RESOURCE	SOURCE	IDENTIFIER
**Antibodies**		
Anti-chicken igY(IgG) (ChIP-qPCR)	Sigma-Aldrich	C2288-#23M4839
Anti-H3K9ac (ChIP-qPCR)	Merck Millipore	06 -942-#2511087-#31636
Anti-H3K4me2 (ChIP-qPCR)	Abcam	Ab32356-#GR253788-9
Anti-H3K4me2 (ChIP-seq)	Merck Millipore	07-030-#2309072
Anti-H3K4me3 (ChIP-qPCR and ChIP-seq)	Diagenode	030-050-#1-#3
Anti-H3K27ac (ChIP-qPCR)	Abcam	Ab4729-#GR251958-1
Anti-H3K27me3 (ChIP-qPCR)	Merck Millipore	07-449-#3018864-#JBC1865906
Anti-H3K9me3 (ChIP-qPCR)	Merck Millipore	07-442-#3143890-#2724355
Anti-H3K27me3 (Cut & Run)	Diagenode	C15410210 #A2354-002349
Anti-ZFP57 (Cut & Run)	Abcam	Ab45341-#GR3307123-1
Anti-CTCF (Cut & Run)	Diagenode	C15410210 #A2354-002349
Anti-IgG (Cut & Run)	EpiCypher	13-0042K #21337004-13
**Biological samples**		
Dnmt3L$^{-/+}$ mouse embryos	This paper	N/A
B/J and J/B neonate mouse brains	This paper	N/A
B/J and J/B adult mouse brains	This paper	N/A
**Chemicals, peptides, and recombinant proteins**		
TRIzol Reagent	Life Technologies	15596018
DNase	Invitrogen	18068-015
DNase	Promega	RQ1 M6101
RNase DNase I	Life Technologies	1890050
RNasin Plus Ribonuclease Inhibitor	Promega	N261B
Sodium chloride	Fisher Chemical	10616082
TritonX-100	Sigma	T8787
Glycogen	Roche	10901393001
SDS	Invitrogen	15553-035
DpnII	Biolabs	R0543M
NlaIII	Biolabs	R0125S
RNase A	Roche	EN0531
Ligase T4 Roche	Roche	10799009001
Proteinase K	Sigma	P65-56
Phenol-chloroform-isoamyl	Sigma	77617
Nucleic lysis buffer	Sigma	NUC 101-1KT
PIC 7X	Roche	04 693 159 001
Sodium butyrate	Sigma	B5887-5G
Mnase	NEB	M0247S
PBS	ThermoFisher	14190094
BSA	Sigma	A9647
SYBR Green I Master	Roche	04887352001
ESGRO complete plus medium	Millipore	SF001-500P
Matrigel® hESC-Qualified Matrix	Corning	354277

B27	Gibco	17504001
Dynabeads protein A	ThermoFisher	10002D
DMH1	Purified by CCH	N/A
**Critical commercial assays**		
EZ DNA methylation Gold Kit	ZYMO	D5006
RNeasy Mini Kit 50	Qiagen	74104
NucleoSpin RNA Plus	Macherey-Nagel	740984.50
Transcriptase Inverse SuperScript IV	Invitrogen	18090050
Master Mix TaqMan™ PreAmp	Life Technologies	4488593
TruSeq Stranded mRNA	Illumina	20020595
NEBNext® Ultra™ II RNA Library Prep Kit for Illumina	NEB	E7775
CUTANA™ CUT&RUN Kit	Epicypher	14-1048
CUTANA™ CUT&RUN Library Prep Kit	Epicypher	14-1001 & 14-1002
Expand™ Long Template PCR System	Roche	11681842001
DNA Clean & Concentrator Zymo	Qiagen	28104
High Pure PCR Product Purification Kit	Roche	11732676001
Mouse total RNA master panel	Ozyme	636644
Taq-Man PreAmplification Master Mix	Life technologies	4488593
**Deposited data**		
4C-seq for Peg13 DMR, Kcnk9 promoter and PE	This study	GSE244143
ChIP-seq for H3K4me3 and H3K4me2 on ES and NP cells	This study	GSE244144
Cut & Run for H3K27me3, CTCF, ZFP57 on ES and NP cells	This study	GSE244145
RNA-seq on ES and NP cells	This study	GSE244146
**Datasets Reanalyzed**		
Hi-C data for ESCs	*Bonev et al., 2017*	GSM2533818, GSM2533819, GSM2533820, GSM2533821
Hi-C data for in vivo NPC	*Bonev et al., 2017*	GSM2533835, GSM2533836, GSM2533837, GSM2533838
ATAC-seq for ESCs and ESC-derived neural progenitor cells	*Sood et al., 2020*	GSE155215
ChIP-seq for H3K27ac on frontal cortex from the F1 crosses 129X1/SvJ x Cast/EiJ and Cast/EiJ x 129X1/SvJ	*Xie et al., 2012*	GSM751461, GSM751462
RNA-seq on cortex at embryonic day 13.5 from the F1 crosses B6 x JF1 and JF1 x B6	*Bouschet et al., 2017*	GSE58523
WGS of JF1 strain	https://ddbj.nig.ac.jp/	DRP000326, DRP000984
WGBS of ES and NPC	*Stadler et al., 2011*	GSM748786, GSM748788
**Experimental models: Cell lines**		
BJ Hybrid ESC lines	Previously generated *Montibus et al., 2021*	N/A
JB Hybrid ESC lines	Previously generated *Montibus et al., 2021*	N/A
**Experimental models: Organisms/strains**		
C57Bl/6J mouse strain	Charle rivers	000664
JF1/Ms mouse train	Provided by "Plateforme Animalerie Paris-Orsay Centre de Recherche-Institut Curie, France"	N/A

Dnmt3L$^{-/-}$ mouse strain	Previously generated *Hata et al., 2002*	N/A
**Oligonucleotides**		
Primers for PCR, qRT-PCR, ChIP, Bisulfite analysis and 4C	In this paper	Table S1
**Web resources, Software and algorithms**		
bamCoverage	https://gensoft.pasteur.fr/docs/deepTools/3.4.1/content/installation.html	N/A
bcftools	http://www.htslib.org/download/	N/A
bedGraphToBigWig	http://hgdownload.soe.ucsc.edu/admin/exe/linux.x86_64/	N/A
Biostrings	https://bioconductor.org/	N/A
Bismark	https://www.bioinformatics.babraham.ac.uk/projects/bismark/	N/A
bowtie2	https://bowtie-bio.sourceforge.net/bowtie2/manual.shtml	N/A
CutAdapt	https://cutadapt.readthedocs.io/en/stable/	N/A
FourCSeq	https://bioconductor.org/	N/A
freebayes	https://github.com/freebayes/freebayes	N/A
GenomicRanges	https://bioconductor.org/	N/A
GraphPad	https://www.graphpad.com/	N/A
HiC-Pro	https://github.com/nservant/HiC-Pro	N/A
HiTC	https://bioconductor.org/	N/A
Jaspar Database	https://jaspar.elixir.no/	
Macs1.4.2	http://liulab.dfci.harvard.edu/MACS/	N/A
Macs2	https://github.com/macs3-project/MACS	N/A
picard	https://broadinstitute.github.io/picard/	N/A
R	https://cran.r-project.org/	N/A
samtools	http://www.htslib.org/download/	N/A
TopHat2	http://ccb.jhu.edu/software/tophat/index.shtml	N/A
TrimGalore	https://github.com/FelixKrueger/TrimGalore	N/A
UCSC	https://genome.ucsc.edu	N/A
vcflib	https://github.com/vcflib/vcflib	N/A
Vista enhancer browser	https://enhancer.lbl.gov/	N/A

Table S2: Details of the primers used in this study

	Transcript analysis			ChIP analysis			Bisulfite analysis			4C analysis		
Locus	Primers 5' → 3'	SNP B6/JF1	SNP mm39 position	Primers 5' → 3'	SNP B6/JF1	SNP mm39 position	Primers 5' → 3'	SNP B6/JF1	SNP mm39 position	Primers 5' → 3'	SNP B6/JF1	SNP mm39 position
Kcnk9	CTCTTCCCTCCTTTCCTGGTTT<br>GGTGCGAGCTTCAGAGAGGA	G/A	chr15:72,383,806	TCGTGTGCGCTACATCTCCT<br>TGTCCAGTGGAAATTCGCCG	T/C	chr15:72,417,814				CCCCATCTCAAACCCTCTAGC<br>CCCCTTCCCCGCTACTTTTC	T/C	chr15:72,419,891
Peg13	CTCACAAGCAGCAGCTATCG<br>CTTTGGATTGCAGCAGGACT	A/C	chr15:72,678,719	CTCTGTGCTAGCGTCTCCAG<br>AGGCACAGAAAAAGCCCAGA	A/G<br>G/A	chr15:72,681,468<br>chr15:72,681,476	ATAGGGTGATGGYGAGT<br>ACCAACTTCRACTCTCCRAA	A/G<br>G/A	chr15:72,681,468<br>chr15:72,681,476	CCCAACGAAGCAACCTTAGA<br>CCCCAGGATTAAGATAACAAAGAG	A/G	chr15:72,681,242
Trappc9	TCCATGCCACGCTTCTGAAT<br>AAGGGCCAAGAATTGCCTCA	G/A	chr15:72,897,886									
PE	CACAATTGCCACAGAGCATT<br>TCCGCTTTGCAGATTAAAGG	G/T	chr15:72,895,020	TGGTGAGGAAAGGAACTGACG<br>GAAAGATGCACAGGCTGCGA	G/C<br>G/A<br>C/G	chr15:72,893,470<br>chr15:72,893,494<br>chr15:72,893,527				GGTGAGAGGGATACTTGGGAATT<br>ATGGTTTCCTTGTAAGAGCTGTG	A/G	chr15:72,892,295
Chrac1	GTTGCATTCCCTCTTGCAGTG<br>AGCGAACATCAAAGGTAGCAC	C/T<br>G/A	chr15:72,965,737<br>chr15:72,965,789									
Ago2	AAACAGCACTGCGTGGTTTC<br>TCCACCCATTTAACGTTTGCT	T/C<br>C/T	chr15:72,970,657<br>chr15:72,970,672									
Gapdh	ACAGTCCATGCCATCACTGCC<br>GCCTGCTTCACCACCTTCTTG											
Gus	GATTCAGATATCCGAGGGAAAGG<br>GCCAACGGAGCAGGTTGA											
Tbp	GCGATTTGCTGCAGTCATCA<br>CAGCTCCCCACCATGTTCTG											
Rpl30pr				AGCACGCCCAAGACAACGTCA<br>TGTGCGGTAGTTGGTTGCTA								
IAP				TATGCCGAGGGTGGTTCTCTA<br>TGCGGCAAAACTTTATTGCTT								
Hoxa3				CATCCGCTCATACCAAGCTTCTGA<br>GCAGGGAGGTAATTGCTGTGGTTT								
Hoxa10				GGTGGTGATGAGGAAGTCCAT<br>ACCCTGGGCTCTAGAGTCTCTTAT								
Trim28				ACGGCGCTAGTGAGTACTGG<br>ATACTCTCGCACAGGCGCAG								
Grb10				TCAGGGTTGCCATGAGAACCAG<br>TAAGCGAAGCAACACAGCCT								

Table S3: Details of the antibodies used in this study

	Provider	Clonality	Reference	Lots	application
**Anti-Chicken IgY (IgG)**	Sigma-Aldrich	Polyclonal	C2288	23M4839	ChIP-qPCR
**anti-H3K9ac**	Merck Millipore	Polyclonal	06 -942	#2511087; #31636	ChIP-qPCR
**anti-H3K4me2**	Abcam	Monoclonal	Ab32356	GR 253788-9	ChIP-qPCR
**anti-H3K4me2**	Merck Millipore	Polyclonal	07-030	#2309072	ChIP-seq
**anti-H3K4me3**	Diagenode	Polyclonal	030-050	#1; #3	ChIP-qPCR and ChIP-seq
**anti-H3K27ac**	Abcam	Polyclonal	Ab4729	GR251958-1	ChIP-qPCR
**anti-H3K27me3**	Merck Millipore	Polyclonal	07-449	#3018864; JBC1865906	ChIP-qPCR
**anti-H3K27me3**	Cell Signaling	Monoclonal	9733	#19	Cut&Run
**anti-H3K9me3**	Merck Millipore	Polyclonal	07-442	#3143890; #2724355	ChIP-qPCR
**anti-ZFP57**	Abcam	Polyclonal	ab45341	GR3307123-1	Cut&Run
**anti-CTCF**	Cel signaling	Monoclonal	3418	#5	Cut&Run
