# Supplementary material for: Biallelic non-productive enhancer-promoter interactions precede imprinted expression of Kcnk9 during mouse neural commitment
Source: HGG Adv. 2024 Jan 30;5(2):100271. doi: 10.1016/j.xhgg.2024.100271 (PMC10869267; doi:10.1016/j.xhgg.2024.100271)
Supplement: Document S2. Article plus supplemental information [file mmc2.pdf]

HGG Advances

ARTICLE

Biallelic non-productive enhancer-promoter interactions precede imprinted expression of *Kcnk9* during mouse neural commitment

Cecilia Rengifo Rojas,[1,7] Jil Cercy,[1,7] Sophie Perillous,[1] Céline Gonthier-Guéret,[1] Bertille Montibus,[1,9] Stéphanie Maupetit-Méhouas,[1] Astrid Espinadel,[1] Marylou Dupré,[1] Charles C. Hong,[2] Kenichiro Hata,[3,4] Kazuhiko Nakabayashi,[3] Antonius Plagge,[5] Tristan Bouschet,[6] Philippe Arnaud,[1,8,10,*] Isabelle Vaillant,[1,8,*] and Franck Court[1,8,*]

Summary

It is only partially understood how constitutive allelic methylation at imprinting control regions (ICRs) interacts with other regulation levels to drive timely parental allele-specific expression along large imprinted domains. The *Peg13-Kcnk9* domain is an imprinted domain with important brain functions. To gain insights into its regulation during neural commitment, we performed an integrative analysis of its allele-specific epigenetic, transcriptomic, and *cis*-spatial organization using a mouse stem cell-based corticogenesis model that recapitulates the control of imprinted gene expression during neurodevelopment. We found that, despite an allelic higher-order chromatin structure associated with the paternally CTCF-bound *Peg13* ICR, enhancer-*Kcnk9* promoter contacts occurred on both alleles, although they were productive only on the maternal allele. This observation challenges the canonical model in which CTCF binding isolates the enhancer and its target gene on either side and suggests a more nuanced role for allelic CTCF binding at some ICRs.

Introduction

The functional specialization of each cell and tissue type, which is crucial in multicellular organisms, is based on their capacity to respond to developmental and environmental cues by generating specific gene expression profiles. The general principles governing this process have been identified. Key regulatory DNA sequences, sequence-specific transcription factors, epigenetic modifications, and the spatial organization of the genome interact to regulate gene expression levels,[1] raising the question of how their coordinated action is orchestrated. In mammals, this question is particularly important for imprinted genes that are expressed in a parent-of-origin-specific manner.

Genomic imprinting is a key developmental process whereby some mammalian genes are expressed by only one allele, depending on their parental origin. Most of the 200 imprinted genes identified to date are involved in crucial biological processes, such as cell proliferation, fetal and placental growth, energy homeostasis, and metabolic adaptation.[2] Genomic imprinting also plays a central role in brain function and behavior, and many imprinted genes are expressed only in neural lineages.[3] Consequently, misregulation of imprinted genes is causally implicated in severe neurobehavioral disorders such as Prader-Willi and Angelman syndromes.

In humans and mice, most imprinted genes are organized in evolutionarily conserved genomic clusters that contain two or more maternally and paternally expressed genes in large regions (up to several megabases in size). In each cluster, allele-specific expression is primarily regulated by DNA methylation at discrete *cis*-acting regulatory elements known as imprinting control regions (ICRs). Each ICR overlaps with a differentially methylated region (DMR) that harbors allelic DNA methylation inherited from the male or female gamete and subsequently maintained throughout development (i.e., germline DMR). The resulting constitutive allelic DNA methylation at ICRs is critical for orchestrating the allele-specific expression along the imprinted domain by influencing a combination of regulatory mechanisms, some of which are tissue specific, leading to the complex and specific spatiotemporal expression pattern of imprinted genes.[2] Specifically, histone modifications, *cis*-spatial organization, and tissue-specific regulatory regions have all been documented

[1]Genetics, Reproduction and Development Institute (iGReD), CNRS, INSERM, Université Clermont Auvergne, Clermont-Ferrand, France; [2]Department of Medicine, University of Maryland School of Medicine, Baltimore, MD, USA; [3]Department of Maternal-Fetal Biology, National Research Institute for Child Health and Development, 2-10-1 Okura, Setagaya, Tokyo 157-8535, Japan; [4]Department of Human Molecular Genetics, Gunma University Graduate School of Medicine 3-39-22 Showa, Maebashi, Gunma 371-8511, Japan; [5]Department of Biochemistry, Cell and Systems Biology, Institute of Systems, Molecular and Integrative Biology, University of Liverpool, Liverpool, UK; [6]Institut de Génomique Fonctionnelle, CNRS, INSERM, Université de Montpellier, Montpellier, France
[7]These authors contributed equally
[8]Senior author
[9]Present address: Department of Medical and Molecular Genetics, King's College, London, UK
[10]Lead contact
*Correspondence: philippe.arnaud@uca.fr (P.A.), isabelle.vaillant@uca.fr (I.V.), franck.court@uca.fr (F.C.)
https://doi.org/10.1016/j.xhgg.2024.100271.
© 2024 The Author(s). This is an open access article under the CC BY license (http://creativecommons.org/licenses/by/4.0/).

to contribute, along with DNA methylation, to this long-range, ICR-mediated, tissue-specific regulation of imprinted domains.[4,5] Several studies have correlated tissue-specific imprinted expression at imprinted genes with tissue-specific differences in histone modifications at their promoter region.[6–9] For example, placenta-specific paternal deposition of the repressive marks di-methylation of lysine 9 on histone H3 (H3K9me2) and tri-methylation of lysine 27 on histone H3 (H3K27me3) contributes to the placenta-specific maternal expression at a subset of genes in the mouse *Kcnq1* domain.[10–12] In addition, at the maternally methylated ICRs, all of which are also promoters, timely developmental loss or gain of the repressive H3K27me3 mark on the paternal allele contributes to the appropriate tissue-specific paternal expression.[13] Allelic methylation at ICRs may also influence long-range chromatin interactions between enhancer and promoters along imprinted domains. Such interactions between regulatory elements and their target genes are facilitated by sub-chromosomal structures called topological associated domains (TADs).[14] A study on the *H19-Igf2* and *Dlk1-Gtl2* domains, which are controlled by a paternally methylated ICR, showed that binding of the methyl-sensitive and boundary protein CTCF to the unmethylated allele of the ICR induces an allele-specific sub-TAD organization that might facilitate the establishment and maintenance of the imprinted transcriptional program.[15] This observation is in line with previous studies showing that allelic methylation and allelic CTCF binding at *H19* ICR are both critical for mediating parent-specific chromatin loops and ensuring timely and allele-specific enhancer-promoter interactions along the *Igf2-H19* domain.[16–18] A recent study showed that allele-specific CTCF binding at a post-implantation DMR (secondary DMR) structures the *Grb10-Ddc* locus to direct proper enhancer-promoter interactions in the developing heart. This further illustrates the interplay between DNA methylation and CTCF binding to control instructive allelic chromatin configurations at imprinted loci.[19] The observation that CTCF binds to the ICRs of various imprinted loci[20] suggests that the allelic chromatin structure may be a commonly used strategy whereby ICRs direct mono-allelic expression along large genomic imprinted domains. However, for most imprinted clusters, this hypothesis has not been formally evaluated.

Altogether, these data highlight that deciphering how constitutive allelic DNA methylation at ICRs can direct tissue- and stage-specific allele-specific expression along imprinted domains requires the simultaneous analysis of the dynamics of multiple layers of regulation during cell identity acquisition. Here, to gain insights into the regulation of the *Peg13-Kcnk9* domain during neural commitment, we precisely monitored the allele-specific epigenetic, transcriptomic, and *cis*-spatial organization using a mouse stem cell-based corticogenesis model that recapitulates the *in vivo* epigenetic control of imprinted gene expression.[21] The *Peg13-Kcnk9* domain is evolutionarily conserved with important functions in the brain. It contains five genes, two of which are imprinted in both humans and mice: the paternally expressed non-coding RNA *PEG13* and the potassium channel gene *KCNK9*, which is maternally expressed specifically in the brain. The other three genes, *TRAPPC9*, *CHRAC1*, and *AGO2*, are not imprinted in humans, whereas they are preferentially expressed by the maternal allele in the mouse brain.[21–27] Mutations in these genes are associated with neurodevelopmental and neurological disorders,[28–31] including the Birk-Barel intellectual disability syndrome, which is caused by maternally inherited *KCNK9* mutations.[32,33]

Little is known about the mechanisms that control expression along this domain. The *Peg13* promoter overlaps with a germline DMR and might be the ICR of this locus.[22–24] A study using human brain tissues suggests that this DMR controls *KCNK9* and *PEG13* imprinted expression through a CTCF-mediated enhancer-blocking activity.[24] However, this model has not been experimentally validated and it is not known whether it explains the imprinted gene expression kinetics along the domain during neural identity acquisition. The integrative analysis of multiple levels of regulation described in this study provides a comprehensive view of the molecular events that take place during the establishment of maternal *Kcnk9* expression in neural commitment. Our main observation challenges the canonical model of CTCF-mediated enhancer-blocking activity and suggests a more nuanced role for allelic CTCF binding at the ICR of this locus.

Material and methods

Details of key reagents and resources are given in Table S1.

Cell culture and embryonic stem cell differentiation

The hybrid male embryonic stem cell (ESC) lines were previously derived from blastocysts obtained from crosses between C57BL/J (B) and JF1 (J) mice[34] and were maintained in gelatin-coated dishes with ESGRO complete plus medium (Millipore, SF001-500P) containing LIF (leukemia inhibitory factor), BMP4 (bone morphogenetic protein 4), and a GSK3-β (glycogen synthase kinase 3β) inhibitor. *In vitro* corticogenesis was performed as previously described,[35] except that ESCs were plated on Matrigel-coated dishes (human ESC-qualified matrix, Corning), and that the defined default medium was supplemented with B27 (without vitamin A, Gibco) to improve cell survival and with 1 μM dorsomorphin homolog 1 (purified by C.C.H.) to promote neurogenesis.[36] Using this protocol, neural precursor cells (NPCs) are the main cell population after 12 days (D12) of *in vitro* corticogenesis.[35]

Material collection

Neonatal and adult brains were obtained from reciprocal crosses of C57BL/6J (B6) and *Mus musculus molossinus* JF1/Ms mice, (B6xJF1) F1 and (JF1xB6) F1 mice, referred to as BJ and JB in the text. *Dnmt3l*$^{-/+}$ mouse embryos were generated by crossing homozygous *Dnmt3l*$^{-/-}$ females (129SvJae-C57BL/6 hybrid genetic background) with wild-type JF1 males (*M. musculus molossinus*). Embryonic day (E) 9.5 *Dnmt3l*$^{-/+}$ embryos were collected from

pregnant dams. Tail DNA was used for genotyping by PCR as previously described.[37]

DNA methylation analysis

DNA extraction and bisulfite sequencing

DNA was extracted as previously described.[38] Bisulfite conversion was performed with the EZ DNA Methylation-Gold Kit from Zymo (ref. D5006), according to the manufacturer's instructions. PCR amplification, cloning, and sequencing were performed as previously described.[38] Details of the primers used are in Table S2.

DNA methylation data mining

ESC and NPC whole-genome bisulfite sequencing (WGBS) data were obtained from GEO DataSets under the accession numbers GEO: GSM748786 and GEO: GSM748788, respectively. The reads were first processed using TrimGalore and then mapped to the mm39 mouse genome using Bismark. Duplicate reads were removed with the script deduplicate_bismark. CpG methylation levels were computed from the selected alignments using bismark_methylation_extractor (–no_header –cutoff 4 –bedgraph) and coverage2cytosine scripts. The output was converted with the bedGraphToBigWig tools to be loaded on the University of California, Santa Cruz (UCSC) Genome Browser. CpG methylation levels of ESCs, NPCs, and frontal cortex in the mm10 genome were obtained from the tracks Stadler 2011 and Lister 2013 of the DNA methylation Hub on UCSC.

Genome production for next-generation sequencing data alignment of hybrid samples

The sequences of the JF1 strain was obtained from the DDBJ database under the accession numbers DDBJ: DRP000326 and DDBJ: DRP000984. Paired-end reads were filtered using the CutAdapt tool to exclude poor quality reads (–minimum-length 101 –pair-filter any -q 20). The remaining reads were mapped to the mm39 genome with bowtie2. Alignments were filtered for poor quality with samtools view (-q 20). Duplicate alignments were excluded using samtools fixmate and markdup (-r). To identify JF1 polymorphisms, the filtered alignments were analyzed using the freebayes tool (-m 20 -q 30 -C 10 -F 0.75) and the output was normalized using bcftools norm. Then, variants were decomposed using the vcflib vcfallelicprimitives (-kg) tool. The resulting vcf file was then processed with the mm39 genome using a custom R script to generate the genomes used for the alignments (library: Biostrings, GenomicRanges). An mm39 genome masked by N at JF1 single-nucleotide polymorphism (SNP) positions was generated. The JF1 genome was reconstructed by converting JF1 SNPs, insertions, and deletions in the mm39 genome. A diploid hybrid genome that consisted, for each chromosome, of the C57BL/6 and JF1 sequences was generated. The hybrid genome has the advantage of taking the JF1 indels into account when determining allelic alignments, but it results in different genomic coordinates for the same element on the C57BL/6 and JF1 genomes. To work with only one reference, a custom R script was written to convert the coordinates of the JF1 alignments into the reference mm39 genome (GenomicRanges).

Expression analysis

RNA extraction

RNA was isolated from frozen cell pellets using TRIzol Reagent (Life Technologies, 15596018), according to the manufacturer's recommendations.

RT-qPCR

After treatment with RNase-free DNase I (Life Technologies, 180868-015), first-strand cDNA was generated by reverse transcription with Superscript-IV (Life Technologies, 18090050) using random primers and 500 ng of RNA. Then, cDNA was amplified by real-time PCR with the SYBR Green mixture (Roche) using a LightCycler R 480II (Roche) apparatus. The relative expression level was quantified with the 2-delta Ct method that gives the fold change variation in gene expression normalized to the geometrical mean of the expression of the housekeeping genes *Gapdh*, *Tbp*, and *Gus*. The primer sequences are in Table S2.

For allelic analysis, for each locus of interest, the parental allele origin of expression was assigned following direct sequencing of the cognate RT-PCR product that encompassed a strain-specific SNP (SNP details in Table S2).

Microfluidic-based quantitative analysis

This analysis was performed using a commercial panel of total RNA (mouse total RNA master panel; Ozyme 636644) obtained from pooled samples isolated from several hundred mouse embryos and adults. Following reverse transcription, as described above, first-strand cDNA was pre-amplified for 14 cycles with the pool of primers used for the RT-qPCR analysis and the Taq-Man PreAmplification Master Mix (Life Technologies, 4488593). RT-qPCR was then performed and validated on Fluidigm 96.96 Dynamic Arrays using the Biomark HD system (Fluidigm) according to the manufacturer's instructions. The relative gene expression was quantified using the 2-delta Ct method, which gives the fold changes in gene expression normalized to the geometrical mean of the expression of the housekeeping genes *Arbp*, *Gapdh*, and *Tbp*. For each condition, the presented data were obtained from two independent experiments, each analyzed in duplicate.

RNA sequencing

Paired-end RNA sequencing (RNA-seq) data were generated using ESCs and NPCs in duplicate for the B6xJF1 and JF1xB6 genetic backgrounds. RNA-seq libraries were prepared with the Illumina TruSeq Stranded mRNA Kit or the NEBNext Ultra II mRNA-Seq Kit and sequenced on an HiSeq4000 or NovaSeq6000 apparatus by IntegraGen according to the manufacturer's protocol. To determine the global and allelic expression, RNA-seq reads were mapped on the mm39 masked genome and hybrid genome, respectively, using TopHat2 and a gene annotation file adapted for these genomes based on the UCSC refGene track (-r 350 –mate-std-dev 250 –library-type fr-firststrand). Alignments were filtered with samtools for mapping quality and reads mapped in proper pairs (view -f 2 -q 20). This step, on the hybrid alignments, allows obtaining allele-specific mapping. The strand-specific coverages of the RNA-seq data were generated using the C57BL/6 and JF1 specific alignments and the global alignments with bamCoverage (–normalizeUsing RPKM –filterRNAstrand forward/reverse) and visualized on the UCSC genome browser. Replicates were overlaid for allelic and strand-specific coverage using the track collection builder tool for genome exploration.

Gene expression data mining

Expression data of cortex from E13.5 B6xJF1 and JF1xB6 embryos were obtained from the GEO: GSE58523 dataset. The RNA-seq treatment was based on the pipeline described above adapted for single-end RNA-seq.

Chromatin immunoprecipitation

ChIP-qPCR

Chromatin immunoprecipitation (ChIP) of native chromatin was performed as described by Brind'Amour et al.[39] using 500,000 cells

per immunoprecipitation. Results presented in this article were obtained from at least three ChIP assays performed using independent chromatin preparations, as indicated in the figure legends. Details of the antisera used can be found in Table S3. Quantitative and allelic analyses were performed as described previously in Maupetit-Méhouas et al.[13] Details of the SNPs and primers used can be found in Table S2.

ChIP sequencing

ChIP sequencing (ChIP-seq) experiments were performed using native chromatin from ESCs and NPCs (for each cell type: n = 1 in the B/J and n = 1 in the J/B background, respectively), as previously described (Le Boiteux et al.[40]). Details of the used antisera are in Table S3. Background precipitation levels were determined by performing mock precipitations with a nonspecific immunoglobulin (Ig) G antiserum (Sigma-Aldrich C2288), and experiments were validated by qPCR on diagnostic regions before sequencing. Library preparation (TruSeq ChIP Sample Preparation) and sequencing on a HiSeq 2500 instrument (Illumina) were performed by MGX (Montpellier GenomiX), according to the manufacturer's recommendations (mean of 40 million single reads per sample). To determine the global and allelic alignments, ChIP-seq reads were mapped using Bowtie2 to the mm39 masked genome and hybrid genome, respectively. Alignments filtering was done with samtools (view -q 20), peaks were called using MACS1.4.2 (–nomodel –shiftsize 73 –pvalue 1e−5), and the coverage was computed with bamCoverage (–normalizeUsing RPKM –extendReads 200 –ignoreDuplicates –binSize 20). The UCSC track collection builder tool was used to overlay allelic coverages for genome exploration.

Cut&Run

Cut&Run (C&R) was performed using the CUTANA CUT&RUN Kit (Epicypher) and non-fixed nuclei from ESCs and NPCs (for each cell type: n = 1 in the B/J and n = 1 in the J/B background, respectively), according to the manufacturer's instructions. The antisera used are listed in Table S3. Briefly, nuclei were isolated from fresh ESCs or NPCs and stored in nuclear extraction buffer at −80°C. After thawing, 500,000 nuclei per reaction were aliquoted and incubated with pre-activated concanavalin A-coated beads at room temperature for 10 min, followed by overnight incubation with 0.5 μg of antibody in buffer containing 0.01% digitonin at 4°C. Then, nuclei bound to concanavalin A-coated beads were permeabilized with a buffer containing 0.01% digitonin and incubated with the pAG-MNase fusion protein at room temperature for 10 min. After washing, chromatin-bound pAG-MNase cleavage was induced by addition of calcium chloride to a final concentration of 2 mM. After incubation at 4°C for 2 h, the reaction was stopped by addition of stop buffer (containing fragmented genomic *Escherichia coli* DNA as spike-in). Following fragmented DNA purification, Illumina sequencing libraries were prepared from ∼5 ng of purified DNA using the CUTANA CUT&RUN Library Prep Kit (EpiCypher 14–1001 and 14–1002) according to the manufacturer's recommendations. Purified multiplex libraries were diluted to 9 nM concentration (calculated with the Qubit dsDNA HS Assay Kit) and sequenced on a NovaSeq 6000 instrument (Illumina) by IntegraGen SA. Paired-end reads were mapped to the mm39 masked genome and hybrid genome using Bowtie2. Alignment filtering was done with samtools (view -f 2 -q 20), and the coverage was obtained with bamCoverage (global coverage: –scaleFactor "spike-in DNA" –normalizeUsing RPKM –binSize 25; allelic coverage: –binSize 25). The UCSC track collection builder tool was used to overlay allelic coverages for genome exploration. Peaks were called with MACS2 using the Cut&Run control sample (IgG) (callpeak -f BAMPE –keep-dup all).

ChIP-seq and ATAC-seq data mining

Allelic and global mm9 alignments for acetylation of lysine 27 on histone H3 (H3K27ac) in mouse frontal cortex were obtained from the GEO: GSM751461, GSM751462 datasets. These alignments were converted to coverages using an R script (rtracklayer, GenomicRanges) and were visualized on UCSC.

Assay for transposase-accessible chromatin using sequencing (ATAC-seq) data for ESCs and ESC-derived NPCs were obtained from the GEO: GSE155215 DataSet. Paired-end reads were treated with trim_galore (–paired) and then were aligned to the mm39 genome using bowtie2 (–very-sensitive -X 1000). Only properly paired alignments were conserved with samtools (view -f 2) and alignments to mitochondrial sequences and random chromosomes were excluded. PCR duplicates were removed using picard-tools (MarkDuplicates –REMOVE_DUPLICATES true). The coverage was assessed using bamCoverage (–normalizeUsing RPKM –binSize 20) and was visualized on the UCSC genome browser. Peaks were called using macs2 (callpeak -f BAMPE –broad –broad-cutoff 0.05 –keep-dup all).

Circular chromosome conformation capture followed by sequencing

Circular chromosome conformation capture followed by sequencing (4C-seq) experiments were done using ESCs and NPCs from the BxJ genetic background for all viewpoints (for each cell type, *Peg13* DMR n = 2; *Kcnk9* promoter n = 1; putative enhancer [PE] n = 1) and in ESCs from the JxB genetic background for the *Peg13* DMR viewpoint (n = 1). The primers used for each viewpoint can be found in Table S1. 4C template preparation was carried out as previously described[41] with some modifications. Briefly, 1×10^7 cell suspensions were cross-linked with formaldehyde (final concentration 2%) for 10 min. After cell lysis and permeabilization with SDS and Triton X-100, samples were digested with 600 U of *Dpn*II at 37°C in 1× NEBuffer *Dpn*II (4 h with 200 U, overnight with 200 U, and 4 h with 200 U). The restriction enzyme was inactivated with SDS and Triton X-100. The first ligation was performed in a large volume, 7.2 mL of 1× ligase buffer, and with 50 U of T4 DNA ligase, at 18°C overnight. Cross-linking was reversed with 600 μg of proteinase K at 65°C overnight. After phenol/chloroform purification, DNA was digested with 50 U of *Nla*III at 37°C overnight. After phenol/chloroform purification, a second ligation was performed in 14 mL of 1× ligase buffer and with 100 U of T4 DNA ligase at 18°C overnight. The 4C template was concentrated by ethanol precipitation and then purified using the DNA Clean & Concentrator Zymo-25 Kit. To produce a 4C-seq library, 3.2 μg of 4C template was amplified in 16 PCR cycles with viewpoint-specific sequencing primers and 56 U of Expand long template polymerase. PCR reactions were pooled and purified using the High Pure PCR Product Purification Kit. The 4C-seq libraries of the different viewpoints were combined before sequencing on an HiSeq 4000 or NovaSeq 6000 instrument (Illumina) by IntegraGen. Due to the primer design, paired-end reads were used to determine the viewpoint allele and the interacting sequence. To do this, only the expected sequence, corresponding to the viewpoint of the informative reads, was mapped to the mm39 hybrid genome using bowtie2 (–trim5 10 –trim3 "viewpoint specific" –local –very-sensitive-local). Only alignments mapped to the viewpoint coordinates and with a minimal quality were conserved to determine the allelic origin of the viewpoint in the reads (samtools view -q 10 [viewpoint coordinates]). To determine the sequence in interaction, the expected sequence with the *Dpn*II site was mapped to the mm39 masked genome using bowtie2

(–trim5 “viewpoint specific” –trim3 “viewpoint specific” –local –very-sensitive-local). These alignments were filtered for mapping quality (samtools view -q 10) and were split according to the allelic origin of the viewpoint. To construct the allelic interactome, alignments were processed with the FourCSeq Bioconductor package to count the reads mapped exactly to the end of a *Dpn*II fragment and to generate a smoothed rpm normalized coverage. The UCSC track collection builder tool was used to overlay allelic interactome coverages for genome exploration. Based on these read counts, the 4C-ker package[42] was used to identify paternal and maternal interactions (nearBaitAnalysis; k = 8) in replicate experiments. The differential analysis of maternal and paternal interactions was performed using the function “differentialAnalysis” of the 4C-ker package. This function was adapted to handle paired samples and differences were considered significant when the adjusted p value was <0.05.

Re-analysis of high-throughput chromosome conformation capture data

High-throughput chromosome conformation capture (HI-C) data were obtained from the GEO: GSM2533818, GSM2533819, GSM2533820, GSM2533821 DataSets (for ESCs) and from the GEO: GSM2533835, GSM2533836, GSM2533837, GSM2533838 DataSets (for *in vivo* NPCs). These paired-end reads were processed with HiC-Pro[43] with the following parameters: MIN_MAPQ = 20, REFERENCE_GENOME = mm39, GENOME_FRAGMENT = DpnII_resfrag_hg19.bed, LIGATION_SITE = GATC, and BIN_SIZE = 5,000. The produced normalized contact matrix (iced) was used to generate the contact map with the HiTC Bioconductor package.

Statistical analyses

Statistical analyses were performed using GraphPad. The statistical test used for each comparison and the number of independent experiments are indicated in the figure legends.

Results

Kcnk9 gains maternal expression upon neural commitment

Using a microfluidic RT-qPCR approach, we observed, in agreement with allelome studies,[25,27] that *Peg13* was expressed in a wide range of adult mouse tissues and at different development stages, but particularly in brain tissues. Conversely, *Kcnk9* expression was restricted to brain tissues (Figure S1A). Expression analyses in brain tissues from newborn F1 hybrids obtained by crossing C57BL/6J and *M. musculus molossinus* (JF1) mice confirmed that *Peg13* was paternally expressed and *Kcnk9* maternally expressed (Figure 1A). To investigate the mechanisms responsible for this brain-specific imprinted expression, we adapted a stem cell-based corticogenesis model that we have previously shown to recapitulate the *in vivo* epigenetic control of imprinted gene expression[21] to mouse ESC lines we derived from reciprocal crosses of C57BL/6 (B6) and JF1 mice (hereafter, B/J and J/B). Reciprocal crosses allow investigating the parental allele origin using informative SNPs.

We focused on the first 12 days of *in vitro* corticogenesis. During this period, ESCs predominantly differentiated into NPCs, as indicated by the marked downregulation of the pluripotency marker *Pou5f1* and the upregulation of the neural precursor markers *Nestin* and *Pax6* (Figure S1B). RNA-seq and RT-qPCR approaches, performed using B/J- and J/B-derived ESCs, showed that, in ESCs, imprinted expression was restricted to *Peg13*, which showed weak paternal expression. Upon differentiation to NPCs, paternal *Peg13* expression and maternal *Kcnk9* expression increased (Figures 1B, 1C, S1C, and S1D). During this time window, the other three genes of the domain, *Trapc9*, *Ago2*, and *Chrac1*, were biallelically expressed (Figure S1E). A similar expression pattern was observed in primary neural stem cells (neurospheres) from newborn mice.[44] Moreover, re-analysis of RNA-seq data from dorsal telencephalon samples at E13.5[21] demonstrated that, in our corticogenesis model, the imprinted expression pattern at the *Peg13* domain in NPCs, restricted to *Kcnk9* and *Peg13*, recapitulated the pattern observed in embryonic brain *in vivo* (Figures 1B and S1D). Therefore, our ESC-based corticogenesis model to generate NPCs provides a relevant framework to uncover the mechanisms acting at the *Peg13* domain, and particularly those involved in the imprinted expression of *Peg13* and *Knck9*, in neural stem cells and during early brain development.

Maternal DNA methylation at the *Peg13* DMR is required for *Kcnk9* maternal expression

The *Peg13* DMR is the putative ICR proposed to control the imprinted expression of the entire locus. To more formally evaluate the role of allelic DNA methylation in *Kcnk9* expression regulation, we assessed expression of *Peg13* and *Kcnk9* in brain tissue of E9.5 *Dnmt3l*$^{-/+}$ embryos, derived from *Dnmt3l*$^{-/-}$ females in which DNA methylation imprints at ICRs are not established during oogenesis.[37,45] In wild-type embryos, we confirmed the paternal and maternal expression of *Peg13* and *Kcnk9*, respectively (Figure 2). In mutant embryos, the lack of maternal DNA methylation at the *Peg13* DMR (Figure S2) resulted in increased and biallelic expression of *Peg13*, while *Kcnk9* expression was lost (Figure 2). This supports the hypothesis that the *Peg13* DMR is the ICR of the locus and indicates that its maternal DNA methylation is required for the maternal expression of *Kcnk9*.

Changes in imprinted expression upon neural commitment are not associated with changes in epigenetic signatures at the *Peg13* DMR

We then performed an integrative analysis based on allelic ChIP-seq, C&R (both performed with samples from the two reciprocal crosses), and ChIP-qPCR coupled with mining of data obtained using non-allelic ATAC-seq[46] and WGBS[47] to determine whether epigenetic signature changes at the *Knck9* and *Peg13* promoters could explain their expression change upon ESC differentiation into NPCs.

In ESCs, the *Peg13* promoter showed the characteristic feature of an ICR[48]: DNA methylation, the repressive histone mark H3K9me3 and the zinc finger protein ZFP57 associated with its maternal allele, and the permissive

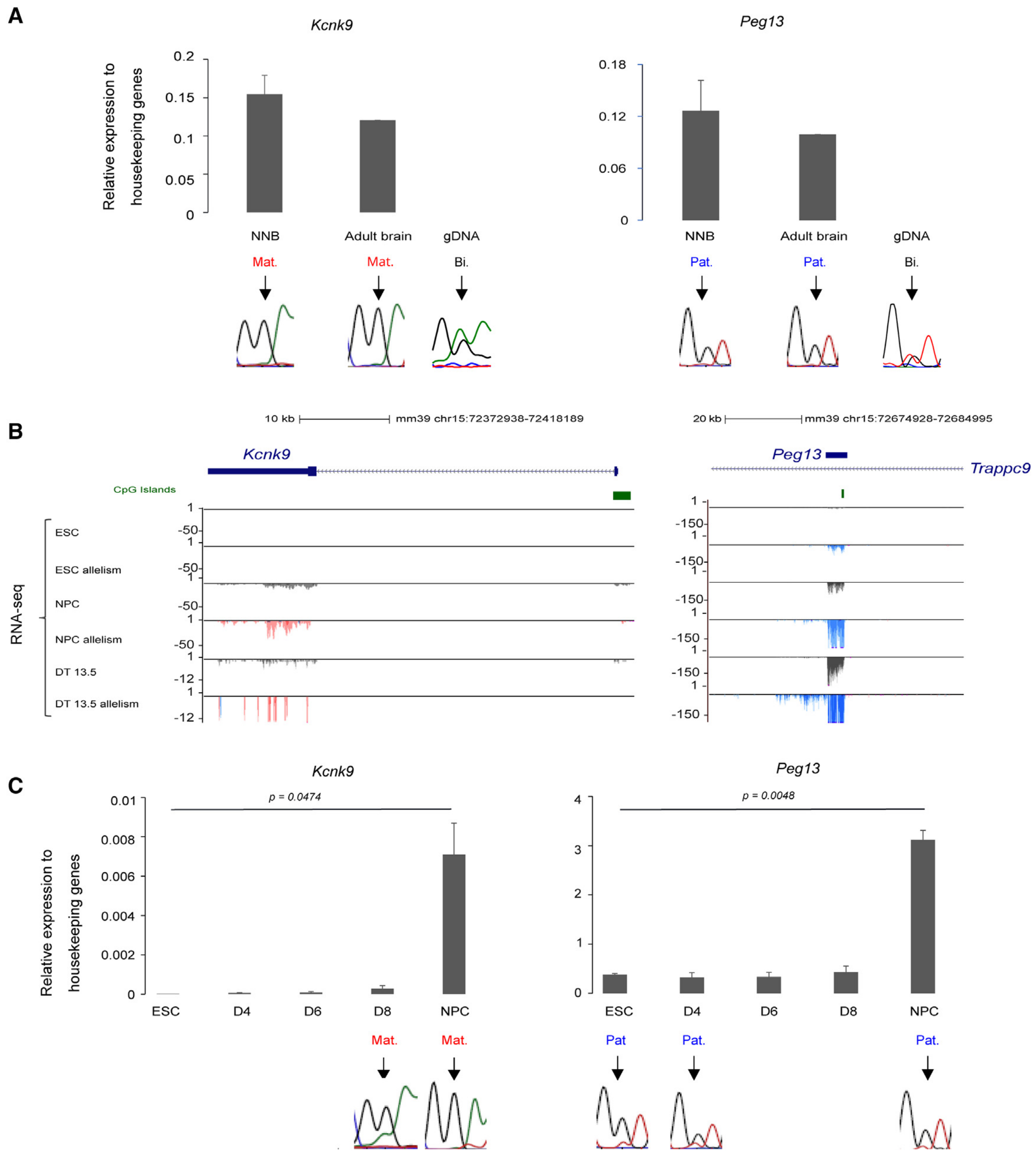

Figure 1. *Kcnk9* and *Peg13* expression dynamics during neural commitment
(A) RT-qPCR analysis of *Kcnk9* and *Peg13* expression levels in the brain of newborn (NBB; n = 4) and adult (n = 2) B/J mice. The parental origin of expression is shown below.
(B) Genome browser view at the *Kcnk9* and *Peg13* loci to show the allelic-oriented RNA-seq signal in B/J ESCs, NPCs, and embryonic dorsal telencephalon (DT); re-analyzed data from Bouschet et al.[21] For each condition, the quantitative and merged parental allelic RNA-seq signals are at the top and bottom, respectively. Maternal and paternal expression levels are shown in red and blue, respectively.
(C) RT-qPCR analysis of *Kcnk9* and *Peg13* expression levels in B/J ESCs (n = 4) and at day 4 (D4; n = 2), D6 (n = 2), D8 (n = 2) of *in vitro* corticogenesis and in NPCs (D12; n = 4). The parental origin of expression is shown below. Statistical significance was determined with the unpaired t test (p values in the figure). In (A) and (C), the results are presented as percentage of expression relative to the geometric mean of the expression of the three housekeeping genes *Gapdh*, *Gus*, and *Tbp*. Data are the mean ± SEM. The parental origin of expression was determined by direct sequencing of sample-specific PCR products based on strain-specific SNPs in the regions analyzed; the SNP presence is visible in the traces obtained using hybrid genomic DNA (gDNA) shown in (A). Representative data are shown.

histone marks di-methylation and tri-methylation of lysine 4 of histone H3 (H3K4me2 and H3K4me3) and H3K27ac associated with its paternal allele (Figures 3, S3A, and S3B). This allelic signature was maintained in NPCs, where the permissive histone marks were more widely distributed along the gene on the paternal allele and also in neonatal mouse brain, without major changes despite *Peg13* upregulation (Figures 1, 3, and S3B).

The *Kcnk9* promoter is in a CpG island that remained unmethylated in both ESCs and NPCs and also in embryonic and adult brain tissues. This indicated that *Kcnk9* expression is not controlled by methylation dynamics at its

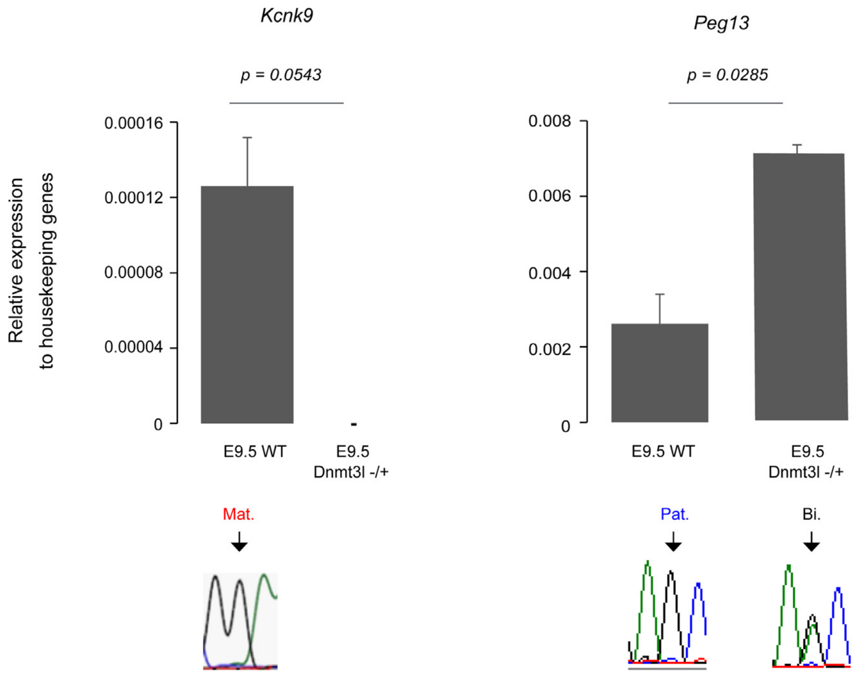

Figure 2. *Kcnk9* expression is lost in E9.5 *Dnmt3l*$^{-/+}$ embryos
RT-qPCR analysis of *Kcnk9* and *Peg13* expression levels in wild-type (n = 2) and *Dnmt3l*$^{-/+}$ (n = 3) E9.5 embryos (head). Statistical significance was determined with the unpaired t test (p values in the figure). Data are the mean ± SEM. The parental origin of expression is shown below. WT, wild type.

promoter (Figures 3A and S3C). Unlike *Peg13*, we did not observe any allelic signature at the *Kcnk9* promoter in ESCs. A broad biallelic H3K27me3 deposition marked the gene body. This repressive mark was associated with the permissive H3K4me2 mark on both alleles of the promoter, forming a bivalent signature that might poise gene expression.[49] In NPCs, H3K27me3 was lost from the gene body, while the promoter retained the bivalent signature, albeit with lower H3K27me3 levels (Figures 3A, 3B, and S3B). ChIP-qPCR, performed using B/J material, also showed that the slight increase of acetylation of lysine 9 on histone H3 (H3K9ac) and H3K27ac, marks associated with active transcription, occurred preferentially on the maternal allele of *Kcnk9* (Figure 3B). This trend was further enhanced in the neonatal brain samples, where H3K9ac, H3K27ac, and H3K4me3 marked the maternal allele of the *Kcnk9* promoter, while the biallelic bivalent signature H3K4me2/H3K27me3 was maintained (Figure 3B).

These observations suggest that upregulation of paternal *Peg13* expression and gain of maternal *Kcnk9* expression upon neural commitment are not driven by changes in the *Peg13* DMR/putative ICR epigenetic signature. Specifically, for *Kcnk9*, maternal expression was induced despite the presence of H3K27me3 at the promoter and was accompanied by biallelic loss of H3K27me3 in the gene body and gain of permissive/activating marks, mainly acetylation, on the maternal promoter reflecting transcriptional activity.

Biallelic interactions between the *Kcnk9* promoter and its putative regulatory region in ESCs precede the maternally biased interaction in NPCs

Besides epigenetic modifications, the higher-order chromatin structure through chromatin looping is another layer of regulation that controls gene expression along imprinted clusters and facilitates enhancer-promoter interactions within TADs. The DNA-binding protein CTCF is a key determinant in the formation of these loops and is frequently found at their base.

Allelic C&R analyses showed that, in both ESCs and NPCs, CTCF bound tightly to the *Peg13* DMR/putative ICR in a paternal-specific manner, whereas it bound to both alleles of the *Kcnk9* promoter and the 3′ edge of its unique intron (Figures 4 and S4).

To determine whether these regions form chromatin loops and to identify putative distant regulatory regions, we performed allelic 4C-seq using the *Peg13* DMR and the *Kcnk9* promoter as viewpoints. While the *Peg13* domain is all contained within a larger TAD (as defined in cortex by Dixon et al.[14]), the signal obtained for the *Peg13* DMR in ESCs was largely restricted to the imprinted domain, from the downstream *Kcnk9* to the upstream *Ago2* gene (Figure S4). Strikingly, in the two reciprocal crosses, contacts were exclusively mediated by the paternal unmethylated *Peg13* DMR, highlighting that paternal CTCF binding promoted higher-order chromatin structure differences between the parental alleles (Figures 4 and S4 and the next section). Although we observed paternal-specific contacts along the entire imprinted domain, we detected significantly stronger paternal signals at *Kcnk9*, centered on the CTCF-bound promoter and the 3′ edge of the intron, and at a biallelic CTCF-bound region in the 5′ part of *Trappc9* (Figures 4 and S4). This second signal peaked in a *Trappc9* intron previously identified as a putative regulatory region that controls the tissue-specific expression of the domain.[44] These paternal-specific contacts were mainly maintained also in NPCs where the interaction with the *Trappc9* intronic putative regulatory region was strengthened (Figures 4 and S4).

The same analysis using the *Kcnk9* promoter as viewpoint confirmed that this promoter interacted with the *Peg13* DMR only on the paternal allele in ESCs and also in NPCs, although more weakly. In addition, the *Kcnk9* promoter interacted with the intronic putative regulatory region in *Trappc9*, from both alleles in ESCs and with a bias from the maternal allele in NPCs (Figure 4).

These results identified a putative regulatory region (the PE), that interacts with the paternal *Peg13* promoter in

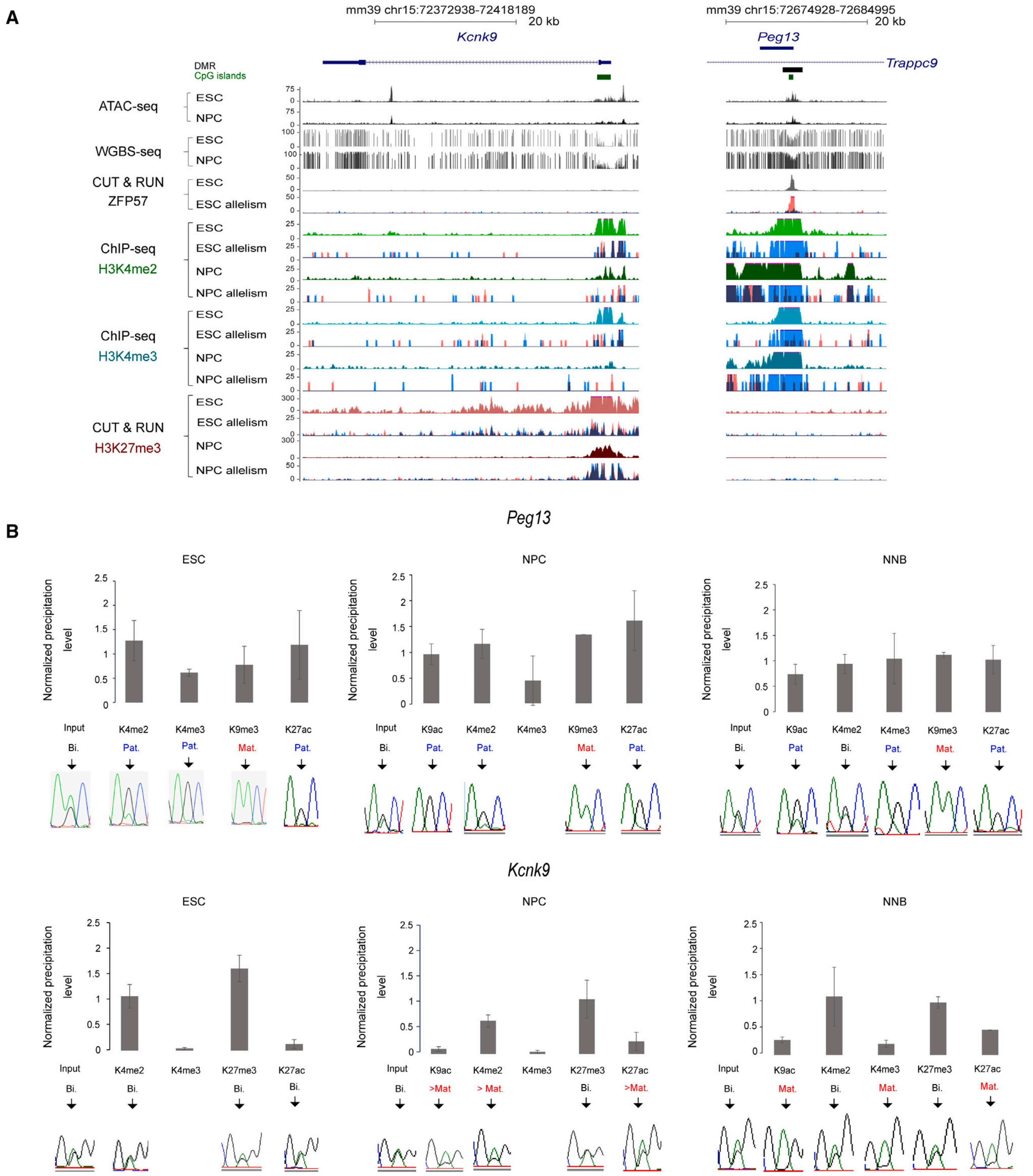

Figure 3. Epigenetic signatures at *Kcnk9* and *Peg13* in ESCs, NPCs, and neonatal brain

(A) Genome Browser view at the *Kcnk9* and *Peg13* loci to show CpG island (CGI) positions and ATAC-seq data, methylation (WGBS), ZPF57, H3K4me2, H3K4me3, and H3K27me3 enrichment in ESCs and NPCs. For ZFP57 and the histone marks, data shown were obtained from B/J material, and the quantitative and the merged parental allelic signals are shown in the upper and lower panels, respectively. Maternal and paternal enrichments are shown in red and blue, respectively.

(B) Chromatin analysis by native ChIP-qPCR to analyze the deposition of the indicated histone marks at the *Peg13* and *Kcnk9* promoters. The precipitation level was normalized to that obtained at the *Rpl30* promoter (for H327ac, H3K4me2, and H3K4me3), the *HoxA3* promoter (for H3K27me3), and IAP (for H3K9me3). For each condition, values are the mean of independent ChIP experiments (n), each performed in duplicate using B/J: ESCs (n = 3), B/J NPCs (n = 3), and B/J neonatal brain (NNB) (n = 5). Data are the mean ± SEM. The allelic distribution of each histone mark was determined by direct sequencing of the sample-specific PCR products containing a strain-specific SNP in the analyzed region; representative data are shown.

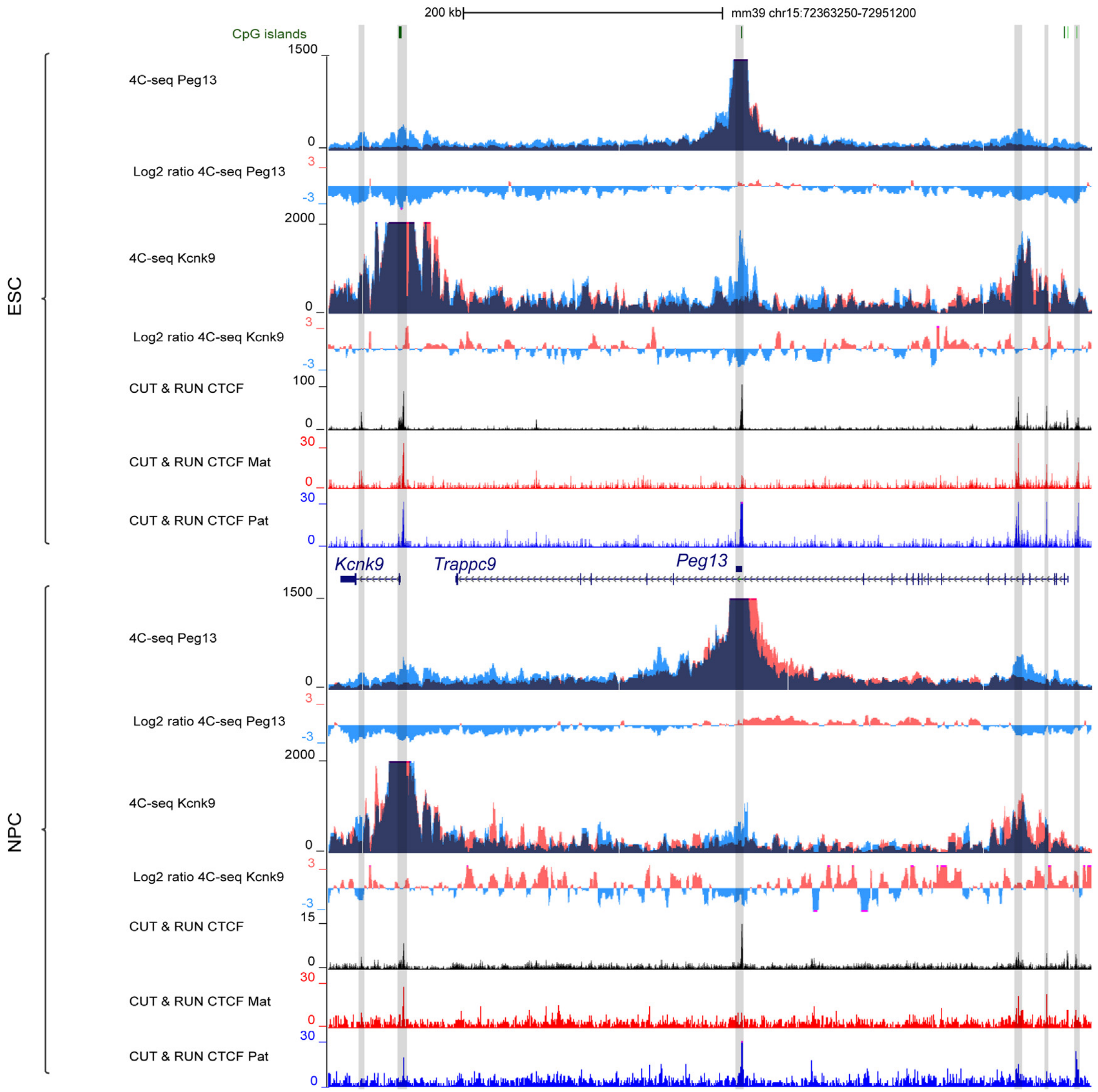

Figure 4. The *Peg13* DMR and *Kcnk9* promoter interact with the same PE in ESCs and NPCs
Genome Browser view of the *Kcnk9-Trappc9* genomic region to show in B/J ESCs (upper panel) and NPCs (lower panel) allelic 4C-seq data, from the *Peg13* DMR and *Kcnk9* promoter viewpoints, and CTCF C&R signals. The 4C-seq data are shown by merging the allelic signals; contacts mediated by the paternal and maternal alleles are shown in blue and red, respectively. The maternal/paternal interaction ratio is shown. CTCF-bound regions are highlighted in gray. Binding is biallelic with the exception of the paternally bound *Peg13* DMR.

ESCs and NPCs, and preferentially, but not exclusively, with the maternal *Kcnk9* promoter in NPCs. Therefore, it is a candidate for regulating the imprinted expression of both genes during neural commitment. However, contrary to expectation, contacts with the *Kcnk9* promoter were already established in ESCs and from both alleles. This suggests that non-productive biallelic contacts between the *Kcnk9* promoter and the putative regulatory region in ESCs precede the maternally biased productive contacts in NPCs.

Contacts between the PE and *Peg13* DMR structure the higher-order chromatin conformation in the *Peg13* domain

To investigate the extent to which the intronic PE influences the chromatin conformation along the *Peg13* domain, we performed allelic 4C-seq using this region as a viewpoint and visualized these data together with allelic 4C-seq data for the *Peg13* DMR and *Kcnk9* promoter. We also re-analyzed high-resolution but non-allelic Hi-C data from ESCs and NPCs *in vivo*.[50] Hi-C data revealed that the *Peg13* imprinted domain resides in two sub-TADs that are conserved in ESCs and NPCs (Figure 5). The centromeric sub-TAD was anchored to CTCF-bound regions in the 5′ part of *Trappc9* and in *Kcnk9*, presumably in the intron (the 5-kb resolution of the Hi-C data did not allow precisely mapping the boundary regions), thus isolating *Kcnk9* and *Peg13* from *Chrac1* and *Ago2*, which are in the telomeric sub-TAD. The *Trappc9* promoter was at the boundary between sub-TADs (Figures 5 and S5). Furthermore, in line with the 4C-seq data, paternal CTCF binding

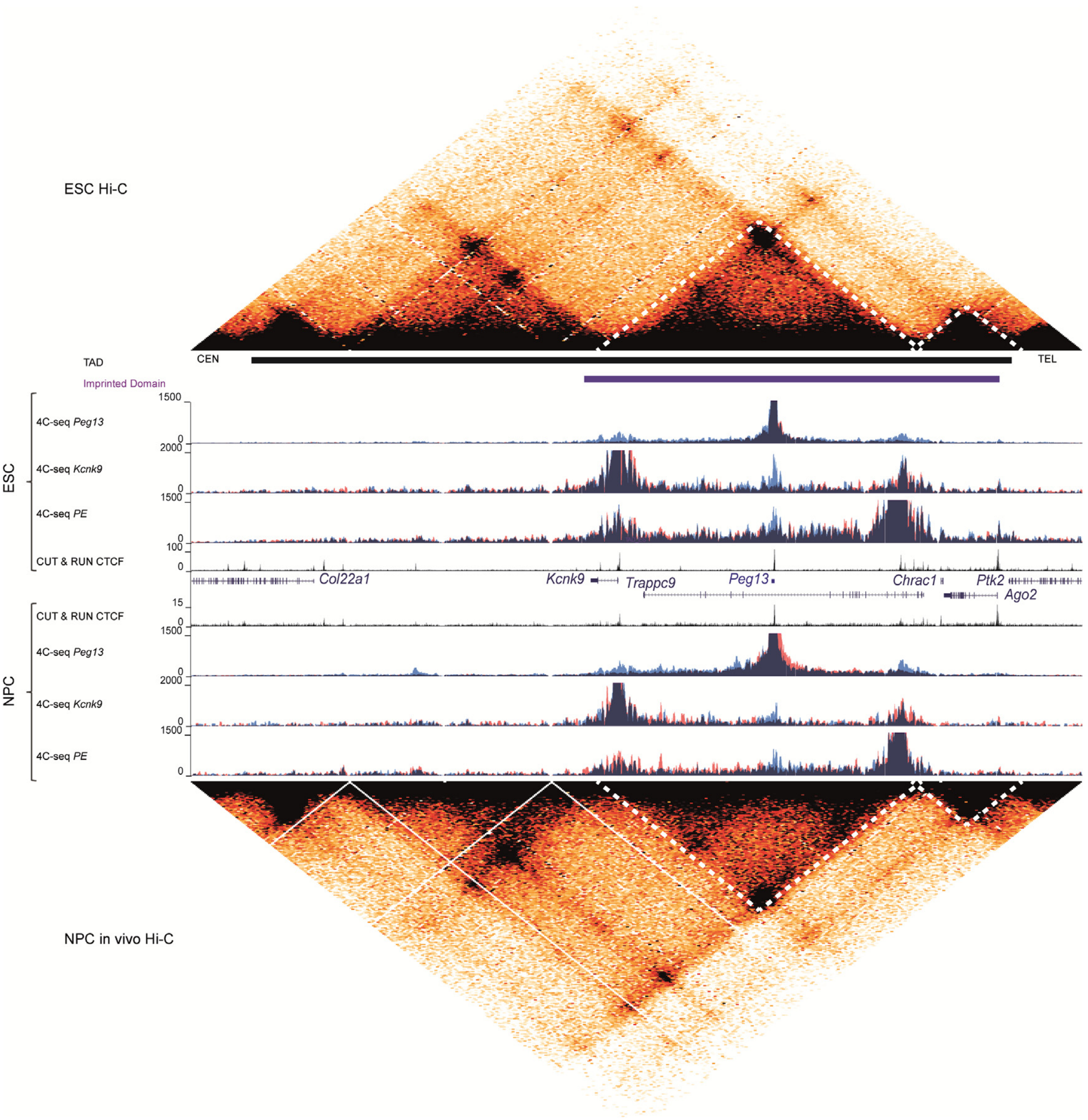

Figure 5. The *Peg13* DMR and PE interactomes structure the higher-order chromatin conformation at the *Peg13* domain
Genome Browser view for the TAD at the *Peg13* domain in ESCs (top panel) and NPCs (bottom panel) to show re-analyzed Hi-C data, allelic 4C-seq from the *Peg13* DMR, *Kcnk9* promoter, and the PE viewpoints and CTCF C&R signals. The 4C-seq data, obtained from B/J material, are shown by merging the allelic signals; contacts mediated by the paternal and maternal alleles are in blue and red, respectively. The sub-TADs that divide the imprinted domain are indicated by a dotted line.

at the *Peg13* DMR subdivided the telomeric sub-TAD into two sub-domains, presumably on the paternal allele only, in a structure maintained in ESCs and NPCs (Figure 5). Notably, the CTCF binding sites identified by the Jaspar database[51] in the *Peg13* DMR were all in the opposite orientation. This suggests that, unlike the majority of loops, which are anchored to pairs of convergent CTCF sites,[52] the loop between the *Peg13* DMR and PE was anchored to a pair of CTCF sites in the same orientation (Figure S5).

Interestingly, in ESCs, this higher-order chromatin structure could not completely isolate sub-TADs or domains from each other. Indeed, the 4C-seq signal obtained for the PE region was mainly, but not entirely, restricted to the centromeric sub-TAD. It was also, albeit to a lesser extent, observed in the telomeric region, including at the *Ago2* promoter (Figure 5). In addition, PE strongly contacted the *Kcnk9* promoter from both alleles, despite the *Peg13* DMR-associated sub-domains on the paternal allele (Figures 5 and S5).

In NPCs, PE contacts were restricted to the centromeric sub-TAD. This coincided with a stronger interaction at the sub-TAD boundary (arrow *b* in Figure S5) that may enhance its insulating capacity (Figures 5 and S5). Along this centromeric sub-TAD, the pattern observed in ESCs remained largely stable also in NPCs, with a conserved, albeit weaker, contact from the paternal allele with the *Peg13* DMR. In addition, the nature of the strong contact with the *Kcnk9* promoter, also observed in the Hi-C data (arrow

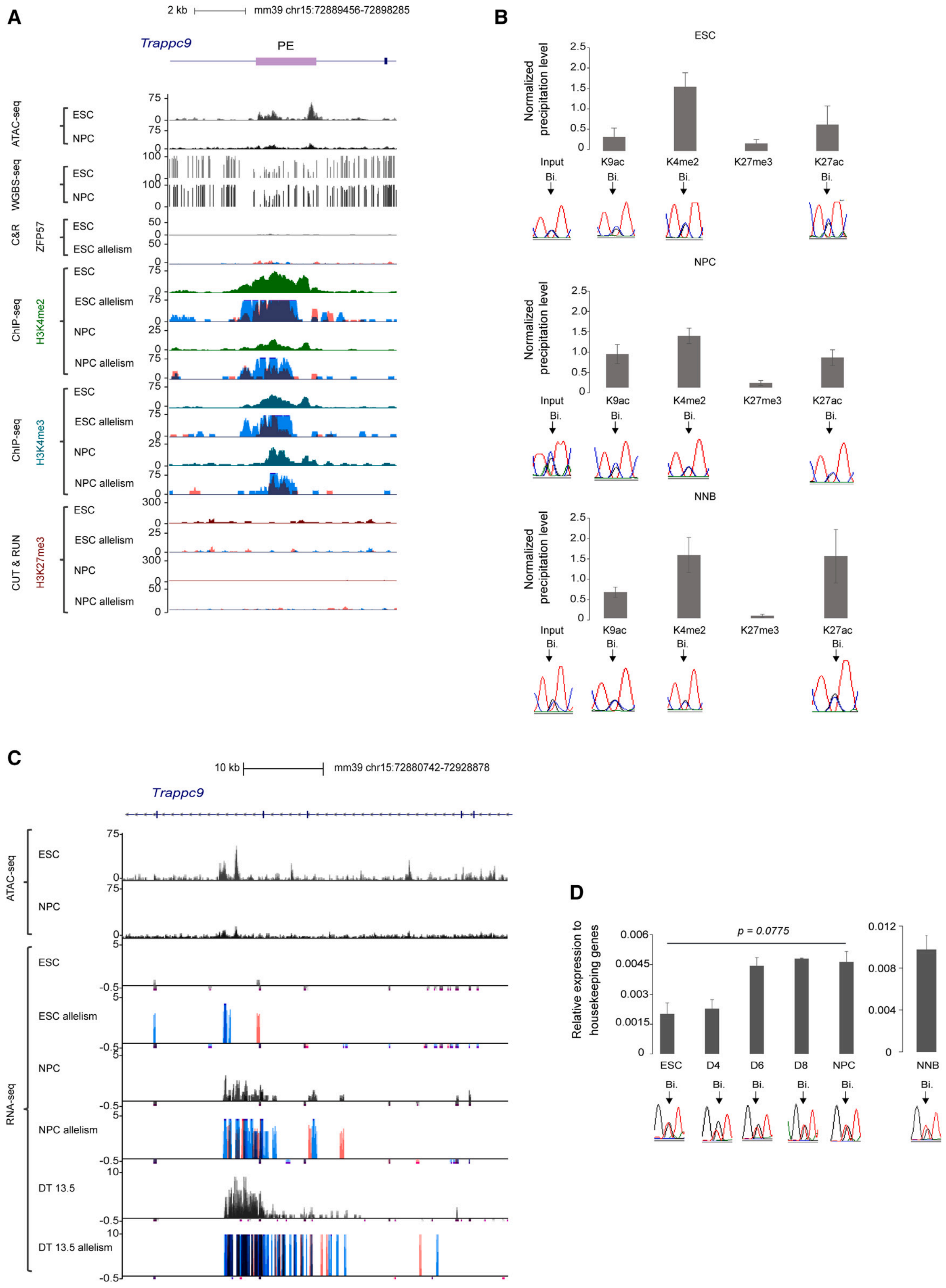

Figure 6. PE molecular signature dynamics during neural commitment

(A) Genome Browser view at the intronic PE region to show ATAC-seq, methylation (WGBS), ZPF57, H3K4me2, H3K4me3, and H3K27me3 enrichment data in ESCs and NPCs. For ZFP57 and the histone marks, data shown were obtained from B/J material; the quantitative and the merged parental allelic signals are shown in the upper and lower panels, respectively. Maternal and paternal enrichments are in red and blue, respectively.

(B) Chromatin analysis following native ChIP-qPCR to analyze the deposition of the indicated histone marks. The precipitation level was normalized to that obtained at the *Rpl30* promoter (for H3K27ac, H3K4me2, and H3K4me3), the *HoxA3* promoter (for H3K27me3), and IAP (for H3K9me3). For each condition, values are the mean of at least three independent ChIP experiments (n), each performed in

(legend continued on next page)

a in Figure S5), changed from biallelic in ESCs to maternally biased, but still biallelic, in NPCs. This change occurred along the entire sub-TAD where we observed preferential maternal and paternal interactions with the regions located on either side of the *Peg13* DMR, respectively (Figure S5).

These data mirror and support those obtained from the 4C-seq data analysis using the *Peg13* DMR and *Kcnk9* promoter as viewpoints (Figures 4 and 5). They highlighted that the *Peg13* DMR organizes the centromeric sub-TAD into two paternal sub-domains that isolate the *Kcnk9* promoter from the PE on the paternal allele. However, this structure might be circumvented in ESCs where PE strongly contacted *Kcnk9* (both alleles), indicating that these contacts precede *Kcnk9* imprinted expression. In NPCs, and consistent with the gain of maternal expression of *Kcnk9*, contacts between PE and the promoter occurred preferentially, although not exclusively, on the maternal allele (Figures 5 and S5).

PE shows features of a biallelically active enhancer in ESCs, NPCs, and neonatal brain

The PE intronic region is one of the enhancers annotated using mouse transgenic experiments[53] with activity in the mouse brain (dataset ID: mm1679 in Vista Enhancer Browser). This finding and our chromatin structure data suggest that this intronic region could be an enhancer for both *Peg13* and *Kcnk9* but that the contacts already established in ESCs are not sufficient to induce maternal *Kcnk9* expression and increased paternal *Peg13* expression.

Therefore, we performed an integrative analysis to determine whether the changes in the molecular signature at this region could account for the change in *Kcnk9* and *Peg13* expression between ESCs and NPCs. Analysis of non-allelic ATAC-seq and WGBS datasets indicated that this region was in an open chromatin configuration, with a strong ATAC-seq signal and DNA methylation depletion in both cell types and brain tissues (Figures 6A and S6). Allelic ChIP-seq, C&R (both performed using samples derived from the two reciprocal crosses), and ChIP-qPCR demonstrated that, in both ESCs and NPCs, as well as in neonatal brain tissue, several permissive/activating marks, including H3K27ac (a signature of active enhancers), were enriched on both alleles, whereas the repressive H3K27me3 mark was absent (Figures 6A and S6B). Besides the histone signature, active enhancers also produce non-coding RNAs called enhancer RNAs (eRNAs).[54] Refined analysis of allelic RNA-seq data identified a biallelically expressed RNA that originated from this region in NPCs and embryonic brain tissues (Figures 6C and S6C). Time-course analysis by RT-qPCR confirmed that this RNA was biallelically expressed and that its expression slightly increased as ESCs differentiated into NPCs and was maintained in neonatal brains (Figure 6D).

Altogether, these observations suggest that the CTCF-bound region in the *Trappc9* intron is a *bona fide* biallelic enhancer that is pre-loaded in an active, but not productive, configuration on the *Kcnk9* promoter in ESCs. Increased activity during ESC differentiation into NPCs and then in neonatal brain is associated with an increase in biallelic eRNA production.

Notably, we also observed that eRNA expression was maternally biased in adult brain (Figure S6D) and by data mining[55] that H3K27ac was enriched on the maternal allele of the PE region in the frontal cortex of adult mice (Figure S6E). This suggests that the enhancer activity can switch from a biallelic to a maternal bias in adult brain.

Discussion

In this study, we wanted to understand the regulation of the *Peg13-Kcnk9* domain during neural commitment. Our mouse stem cell-based corticogenesis model combined with integrative analyses of multiple layers of regulation allowed obtaining a comprehensive view of the molecular events that take place during the establishment of *Kcnk9* maternal expression. We found that, despite the allelic higher-order chromatin structure associated with CTCF, enhancer-*Kcnk9* promoter contacts occurred on both alleles, but they were productive only on the maternal allele. This observation challenges the canonical model in which CTCF binding acts as a chromatin boundary and suggests a more refined role for allelic CTCF binding at this DMR and the resulting allelic chromatin loops at this locus.

The molecular patterns detected at the *Peg13* locus using our stem cell-based corticogenesis model were in agreement with previous *in vivo* observations. This consistency in imprinted gene expression patterns, chromatin signature/conformation, and eRNA production provides additional evidence that our *in vitro* corticogenesis model recapitulates the complex regulations that occur *in vivo* during early brain development.[21] Our observations are also consistent with the results of a study on human brain tissue,[24] thus suggesting that the mechanisms of the *Peg13-Kcnk9* domain regulation are evolutionarily conserved. Therefore, our study provides the basis to

duplicate in B/J ESCs (n = 4), NPCs (n = 3), and NNB (n = 4). The allelic distribution of each histone mark was determined by direct sequencing of the sample-specific PCR products containing a strain-specific SNP in the analyzed region; representative data are shown.
(C) Genome Browser view at the PE region to show the allelic-oriented RNA-seq signal in B/J ESCs, NPCs, and embryonic DT; re-analyzed data from Bouschet et al. For each condition, the quantitative and the merged parental allelic RNA-seq signals are shown in the top and bottom panels, respectively. Maternal and paternal expression levels are in red and blue, respectively.
(D) RT-qPCR analyses to assess PE-associated eRNA expression in B/J ESCs (n = 3), at the day 4 (D4; n = 2), D6 (n = 2), D8 (n = 2), and NPC (D12; n = 3) stages of *in vitro* corticogenesis, and in B/J NNBs (n = 4). Statistical significance was determined with the unpaired t test (p values in the figure). The parental origin of expression is shown in the lower panel. (B and D) Data are the mean ± SEM.

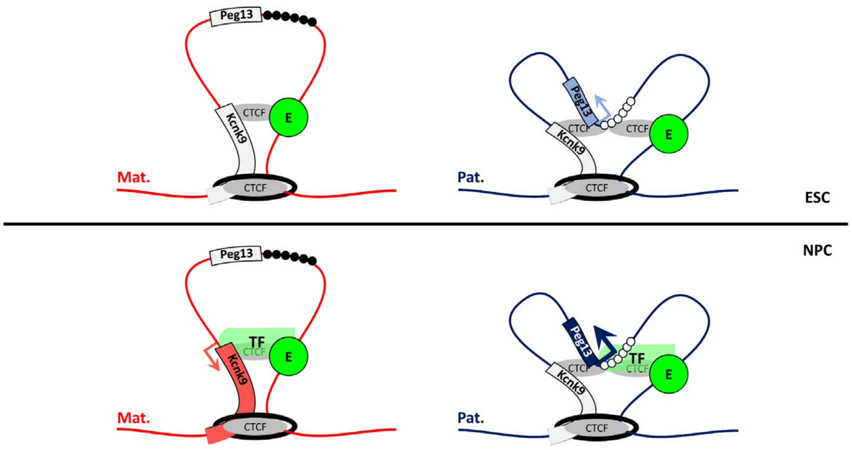

Figure 7. Working model
The sub-TAD anchored to CTCF-bound regions in the *Kcnk9* intron and the 5′ part of *Trappc9* provides a higher-order chromatin structure in which CTCF-anchored chromatin loops lead to a three-way *Kcnk9* promoter-*Peg13* promoter/DMR-enhancer (E) interaction on the paternal allele (Pat.). Due to *Peg13* DMR methylation (black circles), interaction occurs only between the *Kcnk9* promoter and the enhancer on the maternal allele (Mat.). This scaffold is present but not yet informative in ESCs. During ESC differentiation into NPCs, it directs productive contacts and recruitment of *ad hoc* activator transcription factors (TF) at the enhancer. On the paternal allele, the pre-existing three-way interaction provides a structure in which the *Peg13* DMR acts as a physical barrier between the *Kcnk9* promoter and the enhancer, allowing the gain of *Peg13* expression while keeping *Kcnk9* silent. On the maternal allele, the pre-existing interaction between the enhancer and *Kcnk9* promoter induces its maternal expression. The transcription machinery will in turn affect the chromatin structure by strengthening this interaction on the maternal allele as expression increases.

investigate the etiology of neurodevelopmental and neurological disorders associated with this locus. Particularly, in addition to the documented missense *Kcnk9* mutations,[32,33] the enhancer appears to be another target region for mutation and/or epigenetic alteration screening in patients with suspected Birk-Barel syndrome.

The observation that, in ESCs and NPCs, *Kcnk9* and the *Peg13* DMR are located in a different sub-TAD compared with *Trappc9*, *Chrac1*, and *Ago2* provides a framework to explain the absence of imprinting in the last three genes in these cell types and more globally in the developing brain. More studies are required to determine whether and how this higher-order chromatin structure is reorganized later during brain development to allow the *Peg13* DMR to direct the mechanism by which *Trappc9*, *Chrac1*, and *Ago2* switch from biallelic to preferential maternal expression in postnatal brain. However, the recent suggestion that enhancer-promoter interactions may be "memorized" to influence promoter activity later in development[56–58] questions whether the inter-sub-TAD interactions observed between the enhancer and the *Ago2* promoter in ESCs may contribute to instruct imprinted expression at later developmental stages.

Consistent with its germline DMR status,[23] our data suggest that the *Peg13* promoter is the ICR of the locus. Indeed, it exhibits the characteristic allelic molecular feature of an ICR and its maternal methylation is required to control the maternal expression of *Kcnk9*, located approximately 250 kb away. Moreover, it is the only region of the domain that recruits CTCF in a parental allele-specific manner. It is reasonable to assume that the resulting higher-order chromatin structure differences between parental alleles provide a framework in which this ICR can impose the imprinted transcriptional program along the domain, as observed at the *H19-Igf2* and *Dlk1-Gtl2* imprinted loci.[15] However, the underlying mechanism does not follow the canonical model in which parental-specific chromatin loops mediated by CTCF restrict enhancer-promoter interactions to the expressing allele only.[15,17] As previously documented for a minority of enhancer-promoter pairs,[50,58] *Kcnk9* promoter-enhancer interactions are pre-established in ESCs that do not express *Kcnk9* yet. The absence of *Kcnk9* expression at this stage, despite the enhancer active signature, is intriguing. This may be explained by a cell context-dependent dual function of this regulatory element. As observed for other human and mouse regulatory elements,[59,60] it can recruit repressor or activator factors in ESCs and neural cells, respectively. More surprisingly, the enhancer-promoter contacts occur from both alleles, although they are only productive from the maternal allele after differentiation.

These observations suggest an interplay between the pre-existing chromatin structure, the allelic CTCF binding at the *Peg13* DMR, and the transcriptional machinery to shape imprinted expression during neural differentiation. In this model (Figure 7), the sub-TAD anchored to the 5′ part of *Trappc9* and to the *Kcnk9* intron provides a higher-order chromatin structure where CTCF-anchored chromatin loops (not informative at this stage) are formed in ESCs. Upon differentiation and recruitment of activator transcription factors to the enhancer, this structural organization guides productive contacts. On the paternal allele, the pre-existing interactions of the *Peg13* DMR with the enhancer and *Kcnk9* promoter allow the gain of *Peg13* expression and keep *Kcnk9* silent. Specifically, we propose that rather than isolating the enhancer from the promoter, the CTCF-mediated loops induce a three-way *Kcnk9* promoter-*Peg13* promoter-enhancer contact, where promoter competition for transcription factors and/or a physical barrier formed by the *Peg13* DMR between the *Kcnk9* promoter and the enhancer keep *Kcnk9* silent. It has been proposed that this kind of multi-way interaction between enhancers and promoters, facilitated

by the ordered chromatin structure, regulates the temporal expression along the α-globin locus.[61,62] Moreover, there are several examples of an active promoter between an enhancer and another promoter that reduces the activity of the distal promoter.[63–65] The absence of any specific interaction on the paternal allele other than with the *Peg13* DMR and the absence of a strong repressive signature, such as DNA methylation, on the *Kcnk9* promoter rule out the action of a silencer and suggest that this is the main mechanism of *Kcnk9* silencing maintenance. The allelic *Peg13* DMR-associated sub-domains and enhancer-*Kcnk9* promoter interactions we detected on the paternal allele fit with this three-way contact model. On the maternal allele, the pre-existing interaction between the enhancer and the *Kcnk9* promoter induces its maternal expression. Moreover, the associated recruitment of RNAPolII, which promotes enhancer-promoter interactions,[66] will influence the chromatin structure by strengthening enhancer-*Kcnk9* promoter and enhancer-*Peg13* DMR interactions on the maternal and paternal alleles, respectively. These interactions will become stronger as expression increases. This model, which remains to be validated, explains the enhancer allelic specificity despite biallelic interactions with *Kcnk9* and provides an alternative to the canonical isolation model. This model is supported also by the findings of a recent study that overlap and complement our results. Specifically, CRISPR-induced ectopic activation of the *TrappC9* intronic enhancer identified here induced ectopic maternal expression of *Kcnk9* in ESCs. Moreover, their HiC capture data showed that the interaction between this enhancer region and the *Kcnk9* promoter is biallelic in ESCs and maternally biased but biallelic in *in vitro*-induced neurons and brain tissue.[67]

Our data are also in line with those obtained in a recent allelic chromatin conformation analysis in human cells showing that the CTCF-mediated insulator model described at the *IGF2-H19* locus is not applicable to all imprinted loci.[68] Indeed, our observation supports the hypothesis that, although CTCF binds to many ICRs on their unmethylated allele,[69] its function may not be universal at imprinted loci where it may act through different mechanisms.

Data and code availability

- The data generated in this study have been deposited at the GEO data repository under the accession number GEO: GSE244147 and are publicly available as of the date of publication. Original data are available from the lead contact (philippe.arnaud@uca.f) on request.
- This paper does not report original codes.
- Any additional information required to reanalyze the data reported in this paper is available from the lead contact upon request.

Supplemental information

Supplemental information can be found online at https://doi.org/10.1016/j.xhgg.2024.100271.

Acknowledgments

We thank all members of P.A.'s team for critical reading of the manuscript. This research has been financed by the French government IDEX-ISITE initiative 16-IDEX-0001 (CAP 20–25) (Projet Emergence [to P.A. and F.C.], and project Challenge 3-recherche [P.A.]) and Agence Nationale de la Recherche (ANR-23-CE12 CORGI to T.B. and P.A.).

Author contributions

Conceptualization, P.A., I.V., and F.C.; experimental design, P.A. and F.C.; data production, C.R.R., J.C., S.P., C.G.G., B.M., S.M.-M., A.E., M.D., K.H., K.N., A.P., T.B., P.A., I.V., and F.C.; data analysis, C.R.R., J.C., S.P., C.G.G., B.M., S.M.-M., P.A., I.V., and F.C.; resources, C.C.H., K.H., and K.N.; writing – original draft, P.A.; writing – review & editing, P.A., I.V., and F.C.; visualization, C.R.R., J.C., and F.C.; supervision, P.A. and F.C; project administration, P.A.; funding acquisition, P.A. and F.C.

Declaration of interests

The authors declare no competing interests.

Received: October 17, 2023
Accepted: January 23, 2024

Web resources

Web-based resources are provided in Table S1 and in the "web resources, software, and algorithms" section.

References

1. Shukla, V., Cetnarowska, A., Hyldahl, M., and Mandrup, S. (2022). Interplay between regulatory elements and chromatin topology in cellular lineage determination. Trends Genet. *38*, 1048–1061. https://doi.org/10.1016/j.tig.2022.05.011.
2. Tucci, V., Isles, A.R., Kelsey, G., Ferguson-Smith, A.C.; and Erice Imprinting Group (2019). Genomic Imprinting and Physiological Processes in Mammals. Cell *176*, 952–965. https://doi.org/10.1016/j.cell.2019.01.043.
3. Ivanova, E., and Kelsey, G. (2011). Imprinted genes and hypothalamic function. J. Mol. Endocrinol. *47*, R67–R74.
4. Noordermeer, D., and Feil, R. (2020). Differential 3D chromatin organization and gene activity in genomic imprinting. Curr. Opin. Genet. Dev. *61*, 17–24. https://doi.org/10.1016/j.gde.2020.03.004.
5. Hanna, C.W., and Kelsey, G. (2021). Features and mechanisms of canonical and noncanonical genomic imprinting. Genes Dev. *35*, 821–834. https://doi.org/10.1101/gad.348422.121.
6. Yang, Y., Li, T., Vu, T.H., Ulaner, G.A., Hu, J.F., and Hoffman, A.R. (2003). The histone code regulating expression of the imprinted mouse Igf2r gene. Endocrinology *144*, 5658–5670.
7. Lau, J.C.Y., Hanel, M.L., and Wevrick, R. (2004). Tissue-specific and imprinted epigenetic modifications of the human NDN gene. Nucleic Acids Res. *32*, 3376–3382.

8. Li, T., Vu, T.H., Ulaner, G.A., Yang, Y., Hu, J.F., and Hoffman, A.R. (2004). Activating and silencing histone modifications form independent allelic switch regions in the imprinted Gnas gene. Hum. Mol. Genet. *13*, 741–750.
9. Monk, D., Arnaud, P., Apostolidou, S., Hills, F.A., Kelsey, G., Stanier, P., Feil, R., and Moore, G.E. (2006). Limited evolutionary conservation of imprinting in the human placenta. Proc. Natl. Acad. Sci. USA *103*, 6623–6628. https://doi.org/10.1073/pnas.0511031103.
10. Umlauf, D., Goto, Y., Cao, R., Cerqueira, F., Wagschal, A., Zhang, Y., and Feil, R. (2004). Imprinting along the Kcnq1 domain on mouse chromosome 7 involves repressive histone methylation and recruitment of Polycomb group complexes. Nat. Genet. *36*, 1296–1300.
11. Wagschal, A., Sutherland, H.G., Woodfine, K., Henckel, A., Chebli, K., Schulz, R., Oakey, R.J., Bickmore, W.A., and Feil, R. (2008). G9a histone methyltransferase contributes to imprinting in the mouse placenta. Mol. Cell Biol. *28*, 1104–1113.
12. Mager, J., Montgomery, N.D., de Villena, F.P.M., and Magnuson, T. (2003). Genome imprinting regulated by the mouse Polycomb group protein Eed. Nat. Genet. *33*, 502–507.
13. Maupetit-Méhouas, S., Montibus, B., Nury, D., Tayama, C., Wassef, M., Kota, S.K., Fogli, A., Cerqueira Campos, F., Hata, K., Feil, R., et al. (2016). Imprinting control regions (ICRs) are marked by mono-allelic bivalent chromatin when transcriptionally inactive. Nucleic Acids Res. *44*, 621–635. https://doi.org/10.1093/nar/gkv960.
14. Dixon, J.R., Selvaraj, S., Yue, F., Kim, A., Li, Y., Shen, Y., Hu, M., Liu, J.S., and Ren, B. (2012). Topological domains in mammalian genomes identified by analysis of chromatin interactions. Nature *485*, 376–380.
15. Llères, D., Moindrot, B., Pathak, R., Piras, V., Matelot, M., Pignard, B., Marchand, A., Poncelet, M., Perrin, A., Tellier, V., et al. (2019). CTCF modulates allele-specific sub-TAD organization and imprinted gene activity at the mouse Dlk1-Dio3 and Igf2-H19 domains. Genome Biol. *20*, 272. https://doi.org/10.1186/s13059-019-1896-8.
16. Murrell, A., Heeson, S., and Reik, W. (2004). Interaction between differentially methylated regions partitions the imprinted genes Igf2 and H19 into parent-specific chromatin loops. Nat. Genet. *36*, 889–893.
17. Kurukuti, S., Tiwari, V.K., Tavoosidana, G., Pugacheva, E., Murrell, A., Zhao, Z., Lobanenkov, V., Reik, W., and Ohlsson, R. (2006). CTCF binding at the H19 imprinting control region mediates maternally inherited higher-order chromatin conformation to restrict enhancer access to Igf2. Proc. Natl. Acad. Sci. USA *103*, 10684–10689. https://doi.org/10.1073/pnas.0600326103.
18. Court, F., Baniol, M., Hagege, H., Petit, J.S., Lelay-Taha, M.N., Carbonell, F., Weber, M., Cathala, G., and Forne, T. (2011). Long-range chromatin interactions at the mouse Igf2/H19 locus reveal a novel paternally expressed long non-coding RNA. Nucleic Acids Res. *39*, 5893–5906.
19. Juan, A.M., Foong, Y.H., Thorvaldsen, J.L., Lan, Y., Leu, N.A., Rurik, J.G., Li, L., Krapp, C., Rosier, C.L., Epstein, J.A., and Bartolomei, M.S. (2022). Tissue-specific Grb10/Ddc insulator drives allelic architecture for cardiac development. Mol. Cell *82*, 3613–3631.e7. https://doi.org/10.1016/j.molcel.2022.08.021.
20. Lin, S., Ferguson-Smith, A.C., Schultz, R.M., and Bartolomei, M.S. (2011). Nonallelic transcriptional roles of CTCF and cohesins at imprinted loci. Mol. Cell Biol. *31*, 3094–3104. Epub 2011 May 31. https://doi.org/10.1128/MCB.01449-10.
21. Bouschet, T., Dubois, E., Reynès, C., Kota, S.K., Rialle, S., Maupetit-Méhouas, S., Pezet, M., Le Digarcher, A., Nidelet, S., Demolombe, V., et al. (2017). In Vitro Corticogenesis from Embryonic Stem Cells Recapitulates the In Vivo Epigenetic Control of Imprinted Gene Expression. Cereb. Cortex *27*, 2418–2433. https://doi.org/10.1093/cercor/bhw102.
22. Smith, R.J., Dean, W., Konfortova, G., and Kelsey, G. (2003). Identification of novel imprinted genes in a genome-wide screen for maternal methylation. Genome Res. *13*, 558–569. https://doi.org/10.1101/gr.781503.
23. Ruf, N., Bähring, S., Galetzka, D., Pliushch, G., Luft, F.C., Nürnberg, P., Haaf, T., Kelsey, G., and Zechner, U. (2007). Sequence-based bioinformatic prediction and QUASEP identify genomic imprinting of the KCNK9 potassium channel gene in mouse and human. Hum. Mol. Genet. *16*, 2591–2599. https://doi.org/10.1093/hmg/ddm216.
24. Court, F., Camprubi, C., Garcia, C.V., Guillaumet-Adkins, A., Sparago, A., Seruggia, D., Sandoval, J., Esteller, M., Martin-Trujillo, A., Riccio, A., et al. (2014). The PEG13-DMR and brain-specific enhancers dictate imprinted expression within the 8q24 intellectual disability risk locus. Epigenet. Chromatin *7*, 5.
25. Babak, T., DeVeale, B., Tsang, E.K., Zhou, Y., Li, X., Smith, K.S., Kukurba, K.R., Zhang, R., Li, J.B., van der Kooy, D., et al. (2015). Genetic conflict reflected in tissue-specific maps of genomic imprinting in human and mouse. Nat. Genet. *47*, 544–549. https://doi.org/10.1038/ng.3274.
26. Bonthuis, P.J., Huang, W.C., Stacher Hörndli, C.N., Ferris, E., Cheng, T., and Gregg, C. (2015). Noncanonical Genomic Imprinting Effects in Offspring. Cell Rep. *12*, 979–991. https://doi.org/10.1016/j.celrep.2015.07.017.
27. Andergassen, D., Dotter, C.P., Wenzel, D., Sigl, V., Bammer, P.C., Muckenhuber, M., Mayer, D., Kulinski, T.M., Theussl, H.C., Penninger, J.M., et al. (2017). Mapping the mouse Allelome reveals tissue-specific regulation of allelic expression. Elife *6*, e25125. https://doi.org/10.7554/eLife.25125.
28. Liang, Z.S., Cimino, I., Yalcin, B., Raghupathy, N., Vancollie, V.E., Ibarra-Soria, X., Firth, H.V., Rimmington, D., Farooqi, I.S., Lelliott, C.J., et al. (2020). Trappc9 deficiency causes parent-of-origin dependent microcephaly and obesity. PLoS Genet. *16*, e1008916. https://doi.org/10.1371/journal.pgen.1008916.
29. Wilton, K.M., Gunderson, L.B., Hasadsri, L., Wood, C.P., and Schimmenti, L.A. (2020). Profound intellectual disability caused by homozygous TRAPPC9 pathogenic variant in a man from Malta. Mol. Genet. Genomic Med. *8*, e1211. https://doi.org/10.1002/mgg3.1211.
30. Aslanger, A.D., Goncu, B., Duzenli, O.F., Yucesan, E., Sengenc, E., and Yesil, G. (2022). Biallelic loss of TRAPPC9 function links vesicle trafficking pathway to autosomal recessive intellectual disability. J. Hum. Genet. *67*, 279–284. https://doi.org/10.1038/s10038-021-01007-8.
31. Lessel, D., Zeitler, D.M., Reijnders, M.R.F., Kazantsev, A., Hassani Nia, F., Bartholomäus, A., Martens, V., Bruckmann, A., Graus, V., McConkie-Rosell, A., et al. (2020). Germline AGO2 mutations impair RNA interference and human neurological development. Nat. Commun. *11*, 5797. https://doi.org/10.1038/s41467-020-19572-5.
32. Barel, O., Shalev, S.A., Ofir, R., Cohen, A., Zlotogora, J., Shorer, Z., Mazor, G., Finer, G., Khateeb, S., Zilberberg, N., and Birk, O.S (2008). Maternally inherited Birk Barel mental

retardation dysmorphism syndrome caused by a mutation in the genomically imprinted potassium channel KCNK9. Am. J. Hum. Genet. *83*, 193–199. https://doi.org/10.1016/j.ajhg.2008.07.010.
33. Cooper, A., Butto, T., Hammer, N., Jagannath, S., Fend-Guella, D.L., Akhtar, J., Radyushkin, K., Lesage, F., Winter, J., Strand, S., et al. (2020). Inhibition of histone deacetylation rescues phenotype in a mouse model of Birk-Barel intellectual disability syndrome. Nat. Commun. *11*, 480. https://doi.org/10.1038/s41467-019-13918-4.
34. Montibus, B., Cercy, J., Bouschet, T., Charras, A., Maupetit-Méhouas, S., Nury, D., Gonthier-Guéret, C., Chauveau, S., Allegre, N., Chariau, C., et al. (2021). TET3 controls the expression of the H3K27me3 demethylase Kdm6b during neural commitment. Cell. Mol. Life Sci. *78*, 757–768. https://doi.org/10.1007/s00018-020-03541-8.
35. Gaspard, N., Bouschet, T., Herpoel, A., Naeije, G., van den Ameele, J., and Vanderhaeghen, P. (2009). Generation of cortical neurons from mouse embryonic stem cells. Nat. Protoc. *4*, 1454–1463.
36. Neely, M.D., Litt, M.J., Tidball, A.M., Li, G.G., Aboud, A.A., Hopkins, C.R., Chamberlin, R., Hong, C.C., Ess, K.C., and Bowman, A.B. (2012). DMH1, a highly selective small molecule BMP inhibitor promotes neurogenesis of hiPSCs: comparison of PAX6 and SOX1 expression during neural induction. ACS Chem. Neurosci. *3*, 482–491.
37. Hata, K., Okano, M., Lei, H., and Li, E. (2002). Dnmt3L cooperates with the Dnmt3 family of de novo DNA methyltransferases to establish maternal imprints in mice. Development *129*, 1983–1993. https://doi.org/10.1242/dev.129.8.1983.
38. Arnaud, P., Hata, K., Kaneda, M., Li, E., Sasaki, H., Feil, R., and Kelsey, G. (2006). Stochastic imprinting in the progeny of Dnmt3L-/- females. Hum. Mol. Genet. *15*, 589–598. https://doi.org/10.1093/hmg/ddi475.
39. Brind'Amour, J., Liu, S., Hudson, M., Chen, C., Karimi, M.M., and Lorincz, M.C. (2015). An ultra-low-input native ChIP-seq protocol for genome-wide profiling of rare cell populations. Nat. Commun. *6*, 6033. https://doi.org/10.1038/ncomms7033.
40. Le Boiteux, E., Court, F., Guichet, P.O., Vaurs-Barrière, C., Vaillant, I., Chautard, E., Verrelle, P., Costa, B.M., Karayan-Tapon, L., Fogli, A., and Arnaud, P. (2021). Widespread overexpression from the four DNA hypermethylated HOX clusters in aggressive (IDHwt) glioma is associated with H3K27me3 depletion and alternative promoter usage. Mol. Oncol. *15*, 1995–2010. https://doi.org/10.1002/1878-0261.12944.
41. van de Werken, H.J.G., de Vree, P.J.P., Splinter, E., Holwerda, S.J.B., Klous, P., de Wit, E., and de Laat, W. (2012). 4C technology: protocols and data analysis. Methods Enzymol. *513*, 89–112. https://doi.org/10.1016/B978-0-12-391938-0.00004-5.
42. Raviram, R., Rocha, P.P., Müller, C.L., Miraldi, E.R., Badri, S., Fu, Y., Swanzey, E., Proudhon, C., Snetkova, V., Bonneau, R., and Skok, J.A. (2016). 4C-ker: A Method to Reproducibly Identify Genome-Wide Interactions Captured by 4C-Seq Experiments. PLoS Comput. Biol. *12*, e1004780. https://doi.org/10.1371/journal.pcbi.1004780.
43. Servant, N., Varoquaux, N., Lajoie, B.R., Viara, E., Chen, C.J., Vert, J.P., Heard, E., Dekker, J., and Barillot, E. (2015). HiC-Pro: an optimized and flexible pipeline for Hi-C data processing. Genome Biol. *16*, 259. https://doi.org/10.1186/s13059-015-0831-x.
44. Claxton, M., Pulix, M., Seah, M.K.Y., Bernardo, R., Zhou, P., Aljuraysi, S., Liloglou, T., Arnaud, P., Kelsey, G., Messerschmidt, D.M., and Plagge, A. (2022). Variable allelic expression of imprinted genes at the *Peg13, Trappc9, Ago2* cluster in single neural cells. Front. Cell Dev. Biol. *10*, 1022422. https://doi.org/10.3389/fcell.2022.1022422.
45. Bourc'his, D., Xu, G.L., Lin, C.S., Bollman, B., and Bestor, T.H. (2001). Dnmt3L and the establishment of maternal genomic imprints. Science *294*, 2536–2539. https://doi.org/10.1126/science.1065848.
46. Sood, S., Weber, C.M., Hodges, H.C., Krokhotin, A., Shalizi, A., and Crabtree, G.R. (2020). CHD8 dosage regulates transcription in pluripotency and early murine neural differentiation. Proc. Natl. Acad. Sci. USA *117*, 22331–22340. https://doi.org/10.1073/pnas.1921963117.
47. Stadler, M.B., Murr, R., Burger, L., Ivanek, R., Lienert, F., Schöler, A., van Nimwegen, E., Wirbelauer, C., Oakeley, E.J., Gaidatzis, D., et al. (2011). DNA-binding factors shape the mouse methylome at distal regulatory regions. Nature *480*, 490–495. https://doi.org/10.1038/nature10716.
48. Henckel, A., and Arnaud, P. (2010). Genome-wide identification of new imprinted genes. Brief. Funct. Genomics *9*, 304–314. https://doi.org/10.1093/bfgp/elq016.
49. Kumar, D., Cinghu, S., Oldfield, A.J., Yang, P., and Jothi, R. (2021). Decoding the function of bivalent chromatin in development and cancer. Genome Res. *31*, 2170–2184. https://doi.org/10.1101/gr.275736.121.
50. Bonev, B., Mendelson Cohen, N., Szabo, Q., Fritsch, L., Papadopoulos, G.L., Lubling, Y., Xu, X., Lv, X., Hugnot, J.P., Tanay, A., and Cavalli, G. (2017). Multiscale 3D Genome Rewiring during Mouse Neural Development. Cell *171*, 557–572.e24. https://doi.org/10.1016/j.cell.2017.09.043.
51. Castro-Mondragon, J.A., Riudavets-Puig, R., Rauluseviciute, I., Lemma, R.B., Turchi, L., Blanc-Mathieu, R., Lucas, J., Boddie, P., Khan, A., Manosalva Pérez, N., et al. (2022). JASPAR 2022: the 9th release of the open-access database of transcription factor binding profiles. Nucleic Acids Res. *50*, D165–D173. https://doi.org/10.1093/nar/gkab1113.
52. Rao, S.S.P., Huntley, M.H., Durand, N.C., Stamenova, E.K., Bochkov, I.D., Robinson, J.T., Sanborn, A.L., Machol, I., Omer, A.D., Lander, E.S., and Aiden, E.L. (2014). A 3D map of the human genome at kilobase resolution reveals principles of chromatin looping. Cell *159*, 1665–1680. Erratum in: Cell. 2015;162:687-1680. https://doi.org/10.1016/j.cell.2014.11.021.
53. Visel, A., Minovitsky, S., Dubchak, I., and Pennacchio, L.A. (2007). VISTA Enhancer Browser-a database of tissue-specific human enhancers. Nucleic Acids Res. *35*, D88–D92.
54. Kim, T.K., Hemberg, M., Gray, J.M., Costa, A.M., Bear, D.M., Wu, J., Harmin, D.A., Laptewicz, M., Barbara-Haley, K., Kuersten, S., et al. (2010). Widespread transcription at neuronal activity-regulated enhancers. Nature *465*, 182–187. https://doi.org/10.1038/nature09033.
55. Xie, W., Barr, C.L., Kim, A., Yue, F., Lee, A.Y., Eubanks, J., Dempster, E.L., and Ren, B. (2012). Base-resolution analyses of sequence and parent-of-origin dependent DNA methylation in the mouse genome. Cell *148*, 816–831. https://doi.org/10.1016/j.cell.2011.12.035.
56. Xiao, J.Y., Hafner, A., and Boettiger, A.N. (2021). How subtle changes in 3D structure can create large changes in transcription. Elife *10*, e64320. https://doi.org/10.7554/eLife.64320.
57. Wurmser, A., and Basu, S. (2022). Enhancer-Promoter Communication: It's Not Just About Contact. Front. Mol. Biosci. *9*, 867303. https://doi.org/10.3389/fmolb.2022.867303.

58. Ghavi-Helm, Y., Klein, F.A., Pakozdi, T., Ciglar, L., Noordermeer, D., Huber, W., and Furlong, E.E.M. (2014). Enhancer loops appear stable during development and are associated with paused polymerase. Nature *512*, 96–100. https://doi.org/10.1038/nature13417.
59. Huang, D., and Ovcharenko, I. (2022). Enhancer-silencer transitions in the human genome. Genome Res. *32*, 437–448. https://doi.org/10.1101/gr.275992.121.
60. Simeoni, F., Romero-Camarero, I., Camera, F., Amaral, F.M.R., Sinclair, O.J., Papachristou, E.K., Spencer, G.J., Lie-A-Ling, M., Lacaud, G., Wiseman, D.H., et al. (2021). Enhancer recruitment of transcription repressors RUNX1 and TLE3 by mis-expressed FOXC1 blocks differentiation in acute myeloid leukemia. Cell Rep. *36*, 109725.
61. Oudelaar, A.M., Harrold, C.L., Hanssen, L.L.P., Telenius, J.M., Higgs, D.R., and Hughes, J.R. (2019). A revised model for promoter competition based on multi-way chromatin interactions at the α-globin locus. Nat. Commun. *10*, 5412. https://doi.org/10.1038/s41467-019-13404-x.
62. Topfer, S.K., Feng, R., Huang, P., Ly, L.C., Martyn, G.E., Blobel, G.A., Weiss, M.J., Quinlan, K.G.R., and Crossley, M. (2022). Disrupting the adult globin promoter alleviates promoter competition and reactivates fetal globin gene expression. Blood *139*, 2107–2118. https://doi.org/10.1182/blood.2021014205.
63. Bartman, C.R., Hsu, S.C., Hsiung, C.C.S., Raj, A., and Blobel, G.A. (2016). Enhancer Regulation of Transcriptional Bursting Parameters Revealed by Forced Chromatin Looping. Mol. Cell *62*, 237–247. https://doi.org/10.1016/j.molcel.2016.03.007.
64. De Gobbi, M., Viprakasit, V., Hughes, J.R., Fisher, C., Buckle, V.J., Ayyub, H., Gibbons, R.J., Vernimmen, D., Yoshinaga, Y., de Jong, P., et al. (2006). A regulatory SNP causes a human genetic disease by creating a new transcriptional promoter. Science *312*, 1215–1217. https://doi.org/10.1126/science.1126431.
65. Cho, S.W., Xu, J., Sun, R., Mumbach, M.R., Carter, A.C., Chen, Y.G., Yost, K.E., Kim, J., He, J., Nevins, S.A., et al. (2018). Promoter of lncRNA Gene PVT1 Is a Tumor-Suppressor DNA Boundary Element. Cell *173*, 1398–1412.e22. https://doi.org/10.1016/j.cell.2018.03.068.
66. Zhang, S., Übelmesser, N., Barbieri, M., and Papantonis, A. (2023). Enhancer-promoter contact formation requires RNAPII and antagonizes loop extrusion. Nat. Genet. *55*, 832–840. https://doi.org/10.1038/s41588-023-01364-4.
67. Loftus, D., Bae, B., Whilden, C.M., and Whipple, A.J. (2023). Allelic chromatin structure precedes imprinted expression of Kcnk9 during neurogenesis. Genes Dev. *37*, 829–843. https://doi.org/10.1101/gad.350896.123.
68. Richer, S., Tian, Y., Schoenfelder, S., Hurst, L., Murrell, A., and Pisignano, G. (2023). Widespread allele-specific topological domains in the human genome are not confined to imprinted gene clusters. Genome Biol. *24*, 40. https://doi.org/10.1186/s13059-023-02876-2.
69. Prickett, A.R., Barkas, N., McCole, R.B., Hughes, S., Amante, S.M., Schulz, R., and Oakey, R.J. (2013). Genome-wide and parental allele-specific analysis of CTCF and cohesin DNA binding in mouse brain reveals a tissue-specific binding pattern and an association with imprinted differentially methylated regions. Genome Res. *23*, 1624–1635.

HGGA, Volume *5*

Supplemental information

Biallelic non-productive enhancer-promoter interactions precede imprinted expression of *Kcnk9* during mouse neural commitment

Cecilia Rengifo Rojas, Jil Cercy, Sophie Perillous, Céline Gonthier-Guéret, Bertille Montibus, Stéphanie Maupetit-Méhouas, Astrid Espinadel, Marylou Dupré, Charles C. Hong, Kenichiro Hata, Kazuhiko Nakabayashi, Antonius Plagge, Tristan Bouschet, Philippe Arnaud, Isabelle Vaillant, and Franck Court

Supplemental figures and tables :

Figure S1: In the corticogenesis model, expression patterns at the *Peg13* domain recapitulate those observed in embryonic brain *in vivo*

Figure S2: DNA methylation is lost at the *Peg13 DMR* in E9.5 *Dnmt3l* $^{-/+}$ embryos

Figure S3: *Peg13* DMR and *Kcnk9* methylation patterns

Figure S4: Parental allelic 4C-seq signals for the *Peg13* DMR viewpoint in in ESCs and NPCs

Figure S5: The *Peg13* DMR and *PE* interactomes organize the higher-order chromatin conformation at the *Peg13* domain

Figure S6: PE molecular signature dynamics during neural commitment

Table S1: Reagent and Resource

Table S2: Details of the primers used in this study

Table S3: Details of the antibodies used in this study

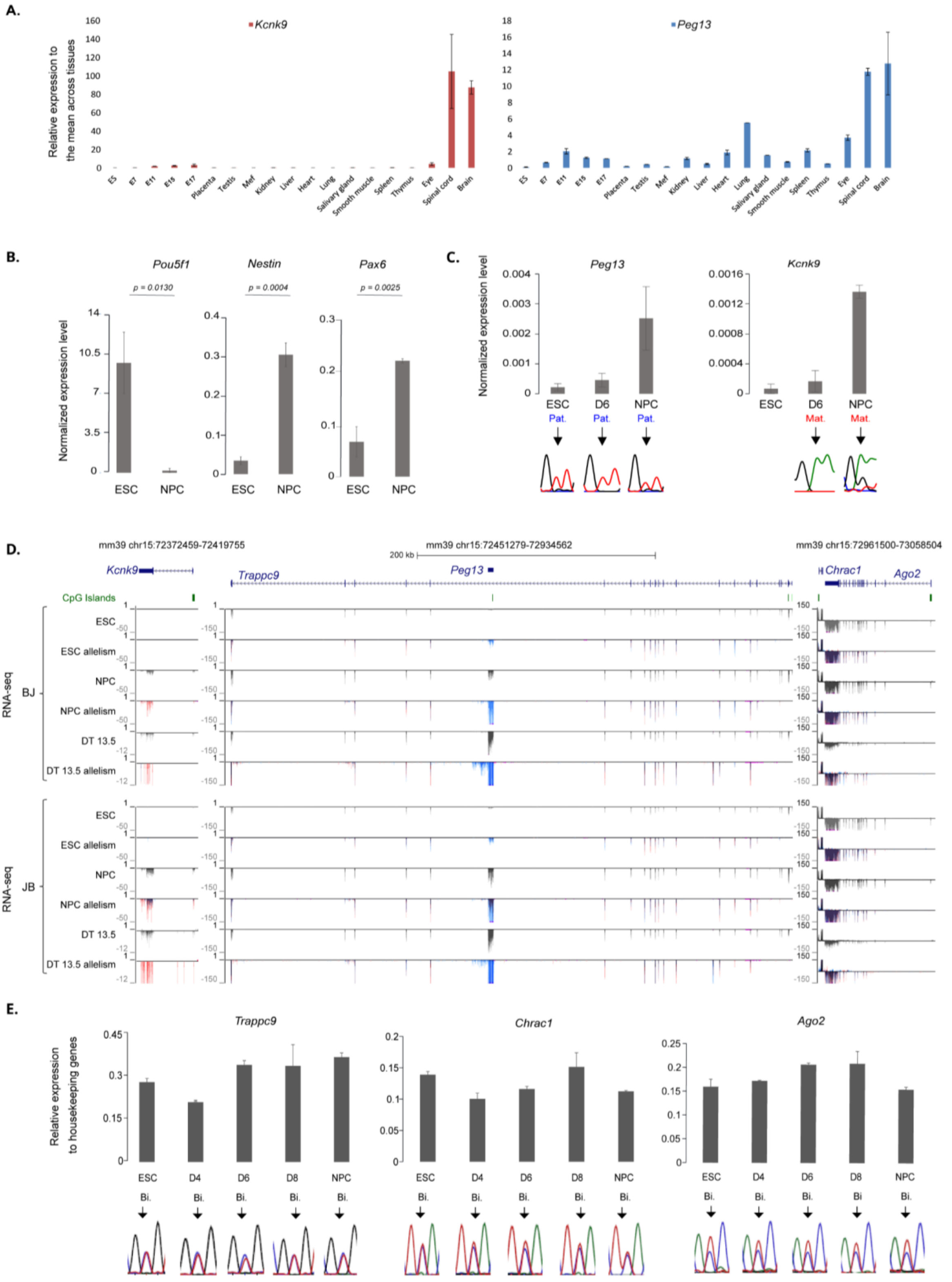

Figure S1: In the corticogenesis model, expression patterns at the *Peg13* domain recapitulate those observed in embryonic brain *in vivo*
A) Microfluidic-based RT-qPCR analysis of *Kcnk9* and *Peg13* expression in the indicated tissues and cell types. Results are presented as the fold enrichment of the mean expression level detected in all tissues, after normalization to the geometric mean of the expression of the three housekeeping genes *Arbp*, *Gapdh* and *Tbp*. Data were from two independent experiments, each analyzed in duplicate. **B)** P*ou5f1*, *Nestin* and *Pax6* expression levels in ESCs (n=6) and in NPCs at day 12 (D12) (n=6) of *in vitro* corticogenesis. P values determined with the unpaired t test. **C)** Quantitative RT-PCR analysis of *Kcnk9* and *Peg13* expression levels in J/B ESCs (n = 3), at D6 (n=3) of *in vitro* corticogenesis and in NPCs (D12; n=3). The parental origin of expression is shown below. **D)** Quantitative RT-PCR analysis of the expression levels of *Trappc9, Chrac1* and *Ago2* in B/J ESCs (*n* = 4) and at the D4 (*n* = 2), D6 (n=2), D8 (*n* = 2), and NPC (D12; *n* = 4) stages of *in vitro* corticogenesis. The parental origin of expression is shown below. **E)** Genome browser view at the *Peg13* domain to show the allelic oriented RNA-seq signal in B/J (upper panel) and J/B (lower panel) ESCs, NPCs and embryonic dorsal telencephalon (DT); re-analyzed data from 21. For each condition the quantitative and the merged allelic RNA-seq signals are shown in the upper and lower panel, respectively. Maternal and paternal expression levels are shown in red and blue, respectively. In **B)** to **D)** results are presented as the percentage of expression relative to the geometric mean of the expression of the three housekeeping genes *Gapdh*, *Gus* and *Tbp*. Data are the mean ± SEM.

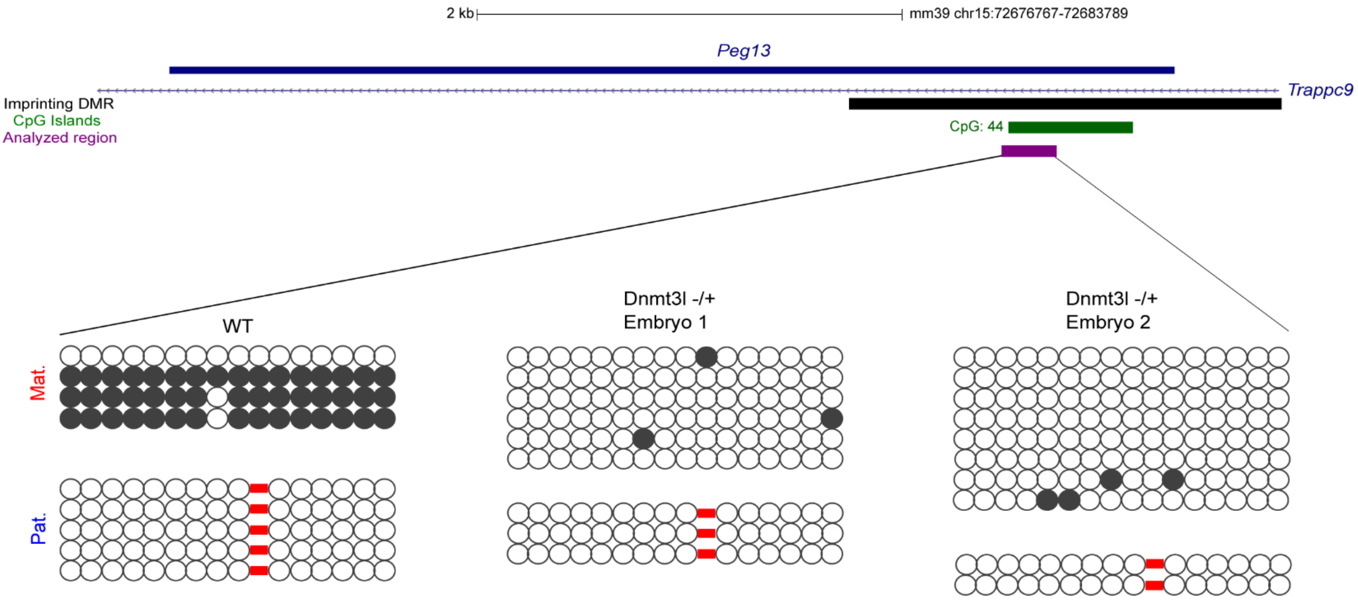

Figure S2: DNA methylation is lost at the *Peg13 DMR* in E9.5 *Dnmt3l* $^{-/+}$ embryos

Map of the mouse *Peg13* locus showing the CpG islands (CGI) as described in the UCSC Genome Browser. The region analyzed by whole-genome bisulfite sequencing is shown in purple. The lower panel shows bisulfite sequencing-derived data from one wild type (WT) and two *Dnmt3l*$^{-/+}$ E9.5 embryos. Each horizontal row of circles represents the CpG dinucleotides on a single chromosome. Solid circles, methylated CpG dinucleotides; open circles, unmethylated CpG dinucleotides. Parental origin (Mat., maternal; Pat., paternal) was determined using strain-specific SNPs. Red rectangles indicate CpGs that are missing due to parental allele specific SNPs.

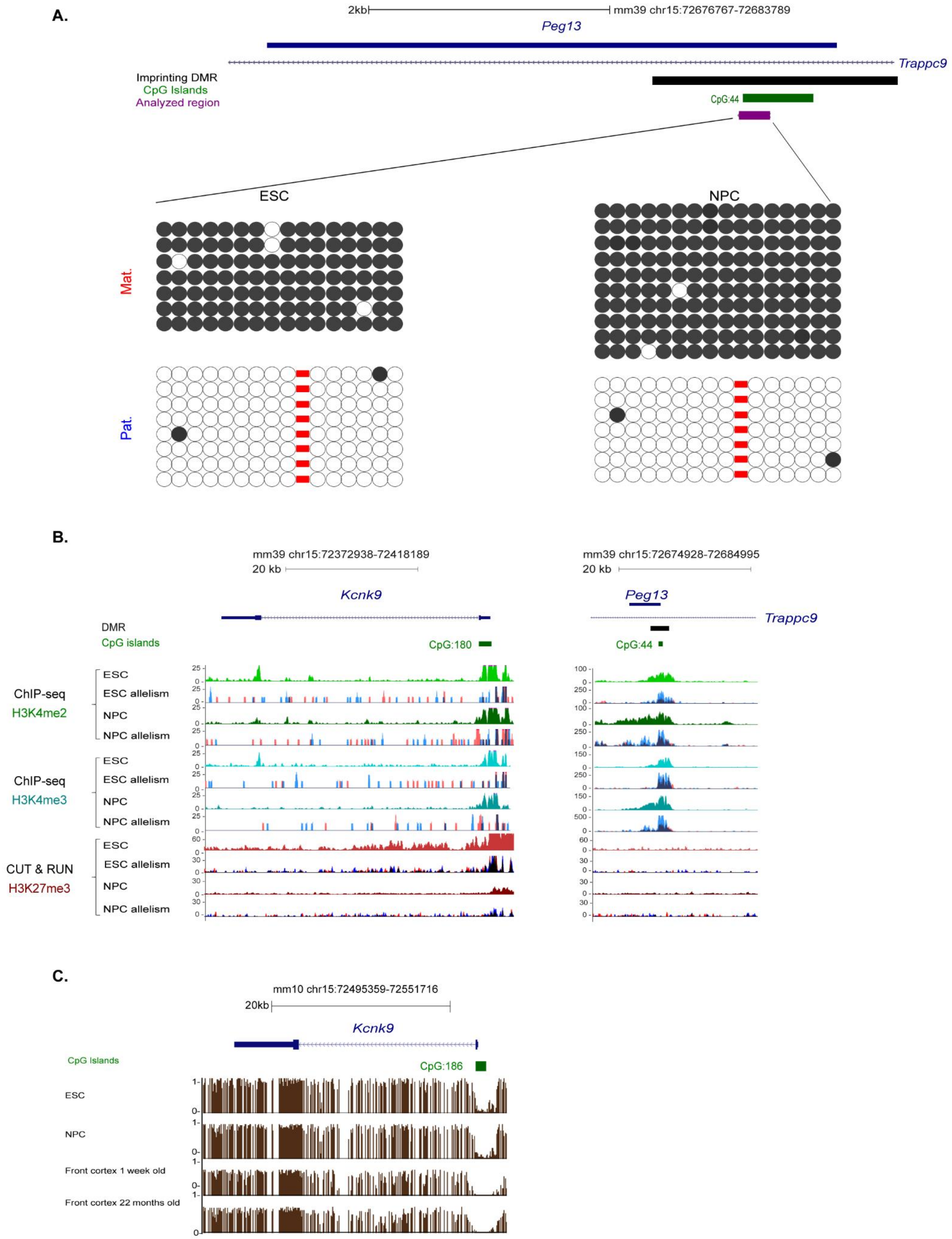

Figure S3: *Peg13* DMR and *Kcnk9* methylation patterns

A) Map of the mouse *Peg13* locus showing the CpG islands (CGI) as described in the UCSC Genome Browser. The region analyzed by bisulfite sequencing is shown in purple. The lower panel shows bisulfite sequencing-derived data from ESCs and NPCs. Each horizontal row of circles represents the CpG dinucleotides on a single chromosome. Solid circles, methylated CpG dinucleotides; open circles, unmethylated CpG dinucleotides. Parental origin (Mat., maternal; Pat., paternal) was determined using strain-specific SNPs. Red rectangles indicate CpGs that are missing due to parental allele specific SNPs. **B)** Genome Browser view at the *Kcnk9* and *Peg13* loci to show CpG island (CGI) positions and H3K4me2, H3K4me3 and H3K27me3 enrichment in J/B ESCs and NPCs. The quantitative and the merged parental allelic signals are shown in the upper and lower panels, respectively. Maternal and paternal enrichments are shown in red and blue, respectively. C) Genome Browser view at the *Kcnk9* locus to show the CpG island (CGI) position and non-allelic WGBS methylation dataset from mouse ESCs, NPCs and frontal cortex.

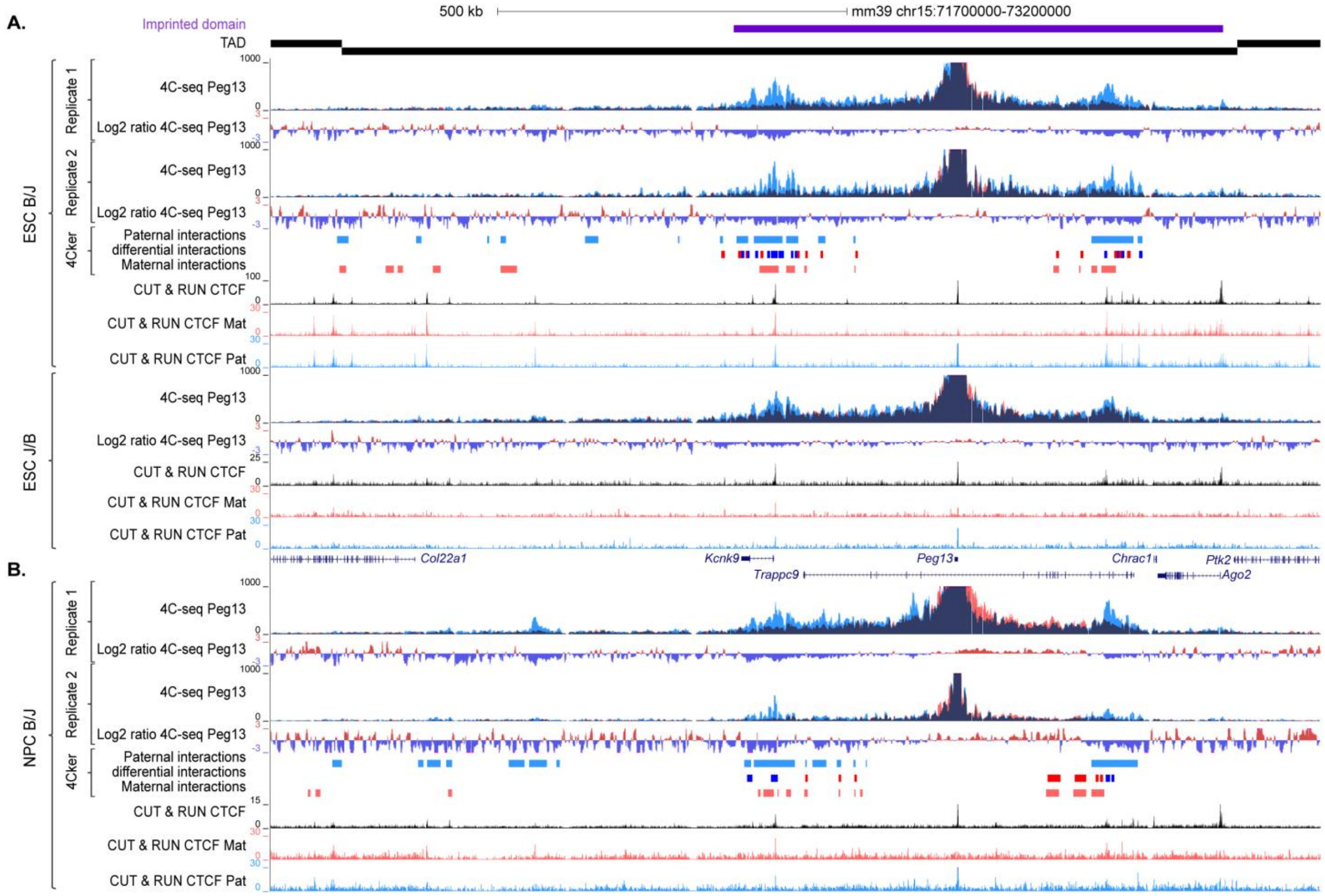

Figure S4: Parental allelic 4C-seq signals for the *Peg13* DMR viewpoint in in ESCs and NPCs

A) Similar parental allelic 4C-seq signals for the *Peg13* DMR viewpoint in B/J and J/B ESCs. Genome browser view at the *Kcnk9 -Trappc9* region to show B/J (upper panel; n=2) and J/B (lower panel, n=1) ESC allelic 4C-seq data for the *Peg13* DMR viewpoint and CTCF C&R signals. **B**) Parental allelic 4C-seq signals for the *Peg13* DMR viewpoint in duplicate (n=2) in B/J NPCs. In **A)** and **B)** 4C-seq data are shown by merging the allelic signals; contacts mediated by the paternal and maternal alleles are shown in blue and red, respectively. The maternal/paternal interaction ratio is indicated. Paternal (blue), maternal (red) and significant ($p<0.05$) allelic differential interactions, identified with the 4Cker tool, are shown. The relative positions of the TAD and the imprinted domain are shown. *Peg13* DMR interactions are largely confined to the imprinted domain within the TAD.

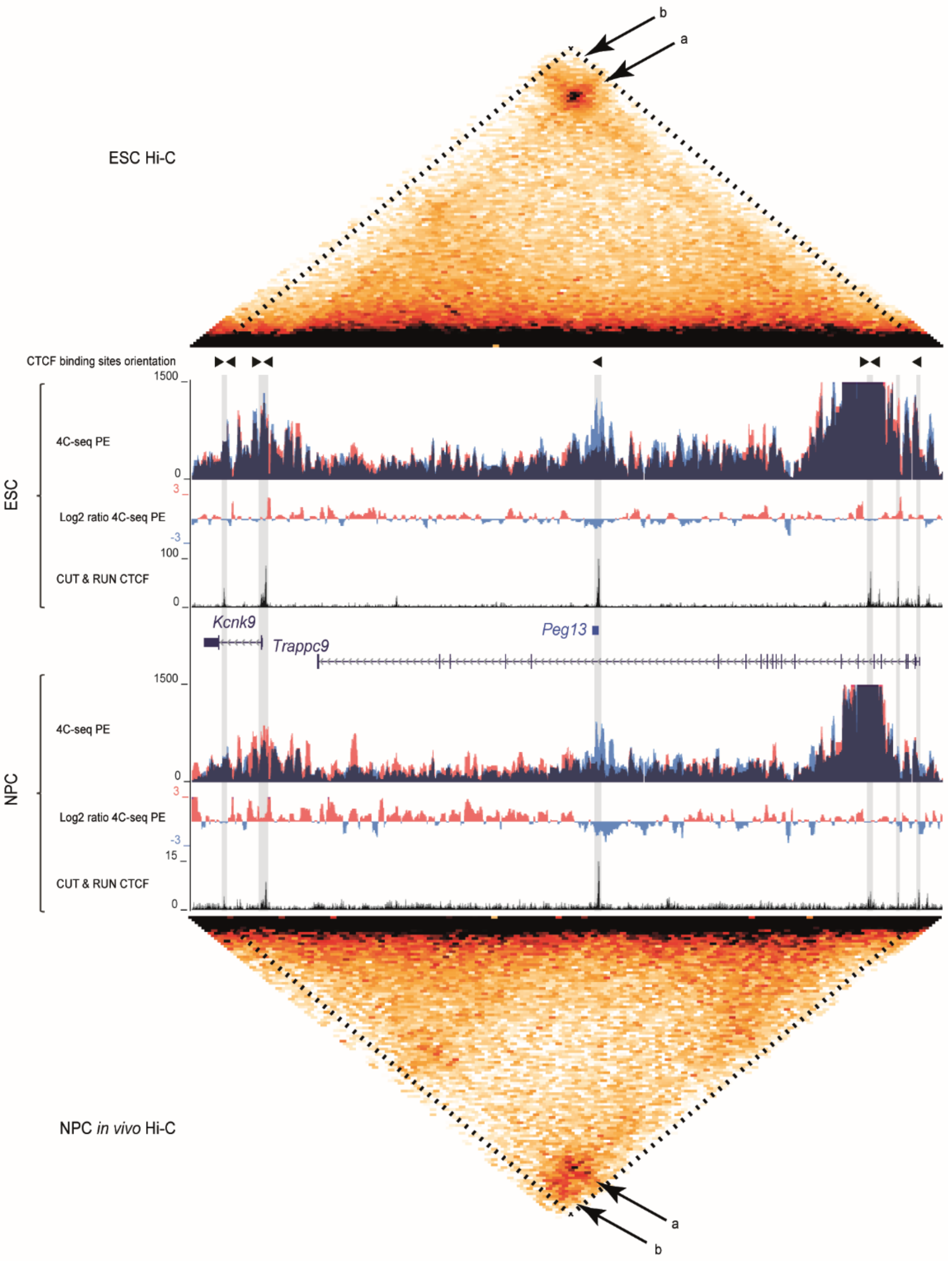

Supp Figure 5: The *Peg13* DMR and *PE* interactomes organize the higher-order chromatin conformation at the *Peg13* domain

Genome Browser view at the *Kcnk9* -*Trappc9* region in ESCs (upper panel) and NPCs (bottom panel); re-analyzed Hi-C data, allelic 4C-seq data for the *Peg13* DMR, *Kcnk9* promoter and the PE viewpoints and CTCF C&R signals. 4C-seq data are presented by merging the allelic signals, contacts mediated by the paternal and maternal alleles are shown in blue and red, respectively. The maternal/paternal interaction ratio is indicated where. The sub-TAD containing the *Kcnk9-Peg13* region is delineated by a dotted line; a and b denote the contact between PE and the *Kcnk9* promoter and between PE and the intronic regions, respectively. The orientation of CTCF binding sites is indicated by arrows.

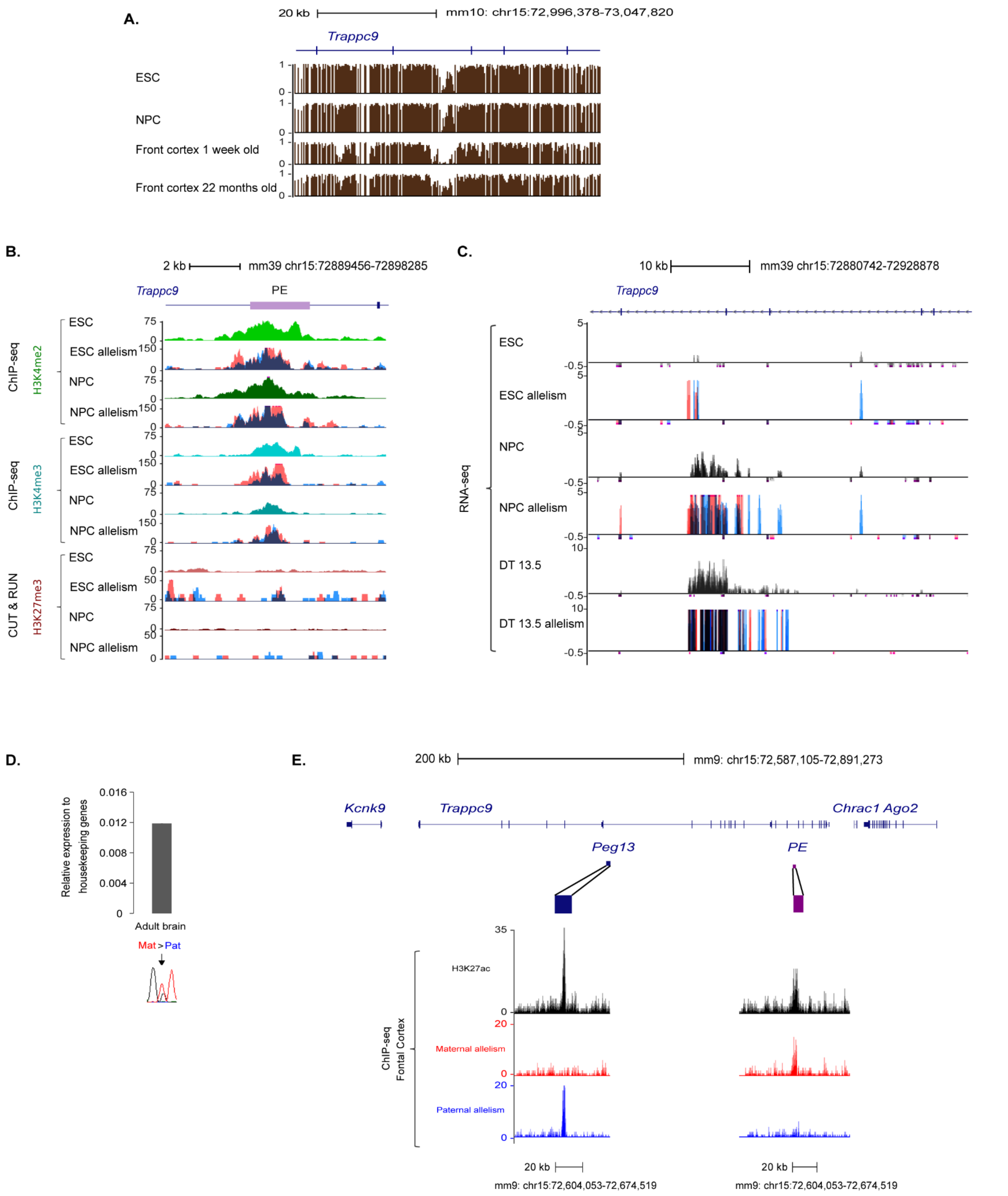

Supp Figure 6: The *Peg13* DMR and *PE* interactomes organize the higher-order chromatin conformation at the *Peg13* domain

A) Genome Browser view at the *PE* region (highlighted in gray) to show non-allelic WGBS methylation data from mouse ESCs, NPCs and frontal cortex. **B)** Genome browser view at the intronic PE region to show ZPF57, H3K4me2, H3K4me3 and H3K27me3 enrichment in J/B ESCs and NPCs. The quantitative and the merged parental allelic signals are shown in the upper and lower panels, respectively. Maternal and paternal enrichments are in red and blue, respectively. **C)** Genome browser view at the PE region to show the allelic oriented RNA-seq signals in J/B ESCs, NPCs and embryonic dorsal telencephalon (DT); re-analyzed data from 21. For each condition, the quantitative and the merged parental allelic RNA-seq signals are shown in the top and bottom panels, respectively. Maternal and paternal expression are in red and blue, respectively. **D)** Quantitative RT-PCR analyses to assess PE-associated eRNA expression in adult brains (n=2). Data are the mean ± SEM. The parental origin of expression is shown below. **E)** Genome Browser view at the *Peg13* and *PE* regions to show allelic H3K27ac enrichment in adult mouse frontal cortex.

**Table S1 :** REAGENT or RESOURCE	SOURCE	IDENTIFIER
**Antibodies**		
Anti-chicken igY(IgG) (ChIP-qPCR)	Sigma-Aldrich	C2288-#23M4839
Anti-H3K9ac (ChIP-qPCR)	Merck Millipore	06 -942-#2511087-#31636
Anti-H3K4me2 (ChIP-qPCR)	Abcam	Ab32356-#GR253788-9
Anti-H3K4me2 (ChIP-seq)	Merck Millipore	07-030-#2309072
Anti-H3K4me3 (ChIP-qPCR and ChIP-seq)	Diagenode	030-050-#1-#3
Anti-H3K27ac (ChIP-qPCR)	Abcam	Ab4729-#GR251958-1
Anti-H3K27me3 (ChIP-qPCR)	Merck Millipore	07-449-#3018864-#JBC1865906
Anti-H3K9me3 (ChIP-qPCR)	Merck Millipore	07-442-#3143890-#2724355
Anti-H3K27me3 (Cut & Run)	Diagenode	C15410210 #A2354-002349
Anti-ZFP57 (Cut & Run)	Abcam	Ab45341-#GR3307123-1
Anti-CTCF (Cut & Run)	Diagenode	C15410210 #A2354-002349
Anti-IgG (Cut & Run)	EpiCypher	13-0042K #21337004-13
**Biological samples**		
Dnmt3L$^{-/+}$ mouse embryos	This paper	N/A
B/J and J/B neonate mouse brains	This paper	N/A
B/J and J/B adult mouse brains	This paper	N/A
**Chemicals, peptides, and recombinant proteins**		
TRIzol Reagent	Life Technologies	15596018
DNase	Invitrogen	18068-015
DNase	Promega	RQ1 M6101
RNase DNase I	Life Technologies	1890050
RNasin Plus Ribonuclease Inhibitor	Promega	N261B
Sodium chloride	Fisher Chemical	10616082
TritonX-100	Sigma	T8787
Glycogen	Roche	10901393001
SDS	Invitrogen	15553-035
DpnII	Biolabs	R0543M
NlaIII	Biolabs	R0125S
RNase A	Roche	EN0531
Ligase T4 Roche	Roche	10799009001
Proteinase K	Sigma	P65-56
Phenol-chloroform-isoamyl	Sigma	77617
Nucleic lysis buffer	Sigma	NUC 101-1KT
PIC 7X	Roche	04 693 159 001
Sodium butyrate	Sigma	B5887-5G
Mnase	NEB	M0247S
PBS	ThermoFisher	14190094
BSA	Sigma	A9647
SYBR Green I Master	Roche	04887352001
ESGRO complete plus medium	Millipore	SF001-500P
Matrigel® hESC-Qualified Matrix	Corning	354277

B27	Gibco	17504001
Dynabeads protein A	ThermoFisher	10002D
DMH1	Purified by CCH	N/A
**Critical commercial assays**		
EZ DNA methylation Gold Kit	ZYMO	D5006
RNeasy Mini Kit 50	Qiagen	74104
NucleoSpin RNA Plus	Macherey-Nagel	740984.50
Transcriptase Inverse SuperScript IV	Invitrogen	18090050
Master Mix TaqMan™ PreAmp	Life Technologies	4488593
TruSeq Stranded mRNA	Illumina	20020595
NEBNext® Ultra™ II RNA Library Prep Kit for Illumina	NEB	E7775
CUTANA™ CUT&RUN Kit	Epicypher	14-1048
CUTANA™ CUT&RUN Library Prep Kit	Epicypher	14-1001 & 14-1002
Expand™ Long Template PCR System	Roche	11681842001
DNA Clean & Concentrator Zymo	Qiagen	28104
High Pure PCR Product Purification Kit	Roche	11732676001
Mouse total RNA master panel	Ozyme	636644
Taq-Man PreAmplification Master Mix	Life technologies	4488593
**Deposited data**		
4C-seq for Peg13 DMR, Kcnk9 promoter and PE	This study	GSE244143
ChIP-seq for H3K4me3 and H3K4me2 on ES and NP cells	This study	GSE244144
Cut & Run for H3K27me3, CTCF, ZFP57 on ES and NP cells	This study	GSE244145
RNA-seq on ES and NP cells	This study	GSE244146
**Datasets Reanalyzed**		
Hi-C data for ESCs	*Bonev et al., 2017*	GSM2533818, GSM2533819, GSM2533820, GSM2533821
Hi-C data for in vivo NPC	*Bonev et al., 2017*	GSM2533835, GSM2533836, GSM2533837, GSM2533838
ATAC-seq for ESCs and ESC-derived neural progenitor cells	*Sood et al., 2020*	GSE155215
ChIP-seq for H3K27ac on frontal cortex from the F1 crosses 129X1/SvJ x Cast/EiJ and Cast/EiJ x 129X1/SvJ	*Xie et al., 2012*	GSM751461, GSM751462
RNA-seq on cortex at embryonic day 13.5 from the F1 crosses B6 x JF1 and JF1 x B6	*Bouschet et al., 2017*	GSE58523
WGS of JF1 strain	https://ddbj.nig.ac.jp/	DRP000326, DRP000984
WGBS of ES and NPC	*Stadler et al., 2011*	GSM748786, GSM748788
**Experimental models: Cell lines**		
BJ Hybrid ESC lines	Previously generated *Montibus et al., 2021*	N/A
JB Hybrid ESC lines	Previously generated *Montibus et al., 2021*	N/A
**Experimental models: Organisms/strains**		
C57Bl/6J mouse strain	Charle rivers	000664
JF1/Ms mouse train	Provided by "Plateforme Animalerie Paris-Orsay Centre de Recherche-Institut Curie, France"	N/A

Dnmt3L$^{-/-}$ mouse strain	Previously generated *Hata et al., 2002*	N/A
**Oligonucleotides**		
Primers for PCR, qRT-PCR, ChIP, Bisulfite analysis and 4C	In this paper	Table S1
**Web resources, Software and algorithms**		
bamCoverage	https://gensoft.pasteur.fr/docs/deepTools/3.4.1/content/installation.html	N/A
bcftools	http://www.htslib.org/download/	N/A
bedGraphToBigWig	http://hgdownload.soe.ucsc.edu/admin/exe/linux.x86_64/	N/A
Biostrings	https://bioconductor.org/	N/A
Bismark	https://www.bioinformatics.babraham.ac.uk/projects/bismark/	N/A
bowtie2	https://bowtie-bio.sourceforge.net/bowtie2/manual.shtml	N/A
CutAdapt	https://cutadapt.readthedocs.io/en/stable/	N/A
FourCSeq	https://bioconductor.org/	N/A
freebayes	https://github.com/freebayes/freebayes	N/A
GenomicRanges	https://bioconductor.org/	N/A
GraphPad	https://www.graphpad.com/	N/A
HiC-Pro	https://github.com/nservant/HiC-Pro	N/A
HiTC	https://bioconductor.org/	N/A
Jaspar Database	https://jaspar.elixir.no/	
Macs1.4.2	http://liulab.dfci.harvard.edu/MACS/	N/A
Macs2	https://github.com/macs3-project/MACS	N/A
picard	https://broadinstitute.github.io/picard/	N/A
R	https://cran.r-project.org/	N/A
samtools	http://www.htslib.org/download/	N/A
TopHat2	http://ccb.jhu.edu/software/tophat/index.shtml	N/A
TrimGalore	https://github.com/FelixKrueger/TrimGalore	N/A
UCSC	https://genome.ucsc.edu	N/A
vcflib	https://github.com/vcflib/vcflib	N/A
Vista enhancer browser	https://enhancer.lbl.gov/	N/A

Table S2: Details of the primers used in this study

	Transcript analysis			ChIP analysis			Bisulfite analysis			4C analysis		
Locus	Primers 5' → 3'	SNP B6/JF1	SNP mm39 position	Primers 5' → 3'	SNP B6/JF1	SNP mm39 position	Primers 5' → 3'	SNP B6/JF1	SNP mm39 position	Primers 5' → 3'	SNP B6/JF1	SNP mm39 position
Kcnk9	CTCTTCCCTCCTTTCCTGGTTT<br>GGTGCGAGCTTCAGAGAGGA	G/A	chr15:72,383,806	TCGTGTGCGCTACATCTCCT<br>TGTCCAGTGGAAATTCGCCG	T/C	chr15:72,417,814				CCCCATCTCAAACCCTCTAGC<br>CCCCTTCCCCGCTACTTTTC	T/C	chr15:72,419,891
Peg13	CTCACAAGCAGCAGCTATCG<br>CTTTGGATTGCAGCAGGACT	A/C	chr15:72,678,719	CTCTGTGCTAGCGTCTCCAG<br>AGGCACAGAAAAAGCCCAGA	A/G<br>G/A	chr15:72,681,468<br>chr15:72,681,476	ATAGGGTGATGGYGAGT<br>ACCAACTTCRACTCTCCRAA	A/G<br>G/A	chr15:72,681,468<br>chr15:72,681,476	CCCAACGAAGCAACCTTAGA<br>CCCCAGGATTAAGATAACAAAGAG	A/G	chr15:72,681,242
Trappc9	TCCATGCCACGCTTCTGAAT<br>AAGGGCCAAGAATTGCCTCA	G/A	chr15:72,897,886									
PE	CACAATTGCCACAGAGCATT<br>TCCGCTTTGCAGATTAAAGG	G/T	chr15:72,895,020	TGGTGAGGAAAGGAACTGACG<br>GAAAGATGCACAGGCTGCGA	G/C<br>G/A<br>C/G	chr15:72,893,470<br>chr15:72,893,494<br>chr15:72,893,527				GGTGAGAGGGATACTTGGGAATT<br>ATGGTTTCCTTGTAAGAGCTGTG	A/G	chr15:72,892,295
Chrac1	GTTGCATTCCCTCTTGCAGTG<br>AGCGAACATCAAAGGTAGCAC	C/T<br>G/A	chr15:72,965,737<br>chr15:72,965,789									
Ago2	AAACAGCACTGCGTGGTTTC<br>TCCACCCATTTAACGTTTGCT	T/C<br>C/T	chr15:72,970,657<br>chr15:72,970,672									
Gapdh	ACAGTCCATGCCATCACTGCC<br>GCCTGCTTCACCACCTTCTTG											
Gus	GATTCAGATATCCGAGGGAAAGG<br>GCCAACGGAGCAGGTTGA											
Tbp	GCGATTTGCTGCAGTCATCA<br>CAGCTCCCCACCATGTTCTG											
Rpl30pr				AGCACGCCCAAGACAACGTCA<br>TGTGCGGTAGTTGGTTGCTA								
IAP				TATGCCGAGGGTGGTTCTCTA<br>TGCGGCAAAACTTTATTGCTT								
Hoxa3				CATCCGCTCATACCAAGCTTCTGA<br>GCAGGGAGGTAATTGCTGTGGTTT								
Hoxa10				GGTGGTGATGAGGAAGTCCAT<br>ACCCTGGGCTCTAGAGTCTCTTAT								
Trim28				ACGGCGCTAGTGAGTACTGG<br>ATACTCTCGCACAGGCGCAG								
Grb10				TCAGGGTTGCCATGAGAACCAG<br>TAAGCGAAGCAACACAGCCT								

Table S3: Details of the antibodies used in this study

	Provider	Clonality	Reference	Lots	application
**Anti-Chicken IgY (IgG)**	Sigma-Aldrich	Polyclonal	C2288	23M4839	ChIP-qPCR
**anti-H3K9ac**	Merck Millipore	Polyclonal	06 -942	#2511087; #31636	ChIP-qPCR
**anti-H3K4me2**	Abcam	Monoclonal	Ab32356	GR 253788-9	ChIP-qPCR
**anti-H3K4me2**	Merck Millipore	Polyclonal	07-030	#2309072	ChIP-seq
**anti-H3K4me3**	Diagenode	Polyclonal	030-050	#1; #3	ChIP-qPCR and ChIP-seq
**anti-H3K27ac**	Abcam	Polyclonal	Ab4729	GR251958-1	ChIP-qPCR
**anti-H3K27me3**	Merck Millipore	Polyclonal	07-449	#3018864; JBC1865906	ChIP-qPCR
**anti-H3K27me3**	Cell Signaling	Monoclonal	9733	#19	Cut&Run
**anti-H3K9me3**	Merck Millipore	Polyclonal	07-442	#3143890; #2724355	ChIP-qPCR
**anti-ZFP57**	Abcam	Polyclonal	ab45341	GR3307123-1	Cut&Run
**anti-CTCF**	Cel signaling	Monoclonal	3418	#5	Cut&Run
